# Bold zebrafish (*Danio rerio*) learn faster in a classical associative learning task

**DOI:** 10.1038/s41598-025-00423-6

**Published:** 2025-05-10

**Authors:** Jamie Corcoran, Levi Storks, Ryan Y. Wong

**Affiliations:** 1 University of Nebraska at Omaha Psychology Department, Omaha, NE USA; 2 University of Nebraska at Omaha Biology Department, Omaha, NE USA; 3 University of Detroit Mercy Biology Department, Detroit, MI US

**Keywords:** Personality, Cognition, Bold, Shy, Associative learning, Behavioural ecology, Cognitive neuroscience

## Abstract

Animals vary between individuals in their ability to learn and some sources of this variation can be the learning process and the individual’s personality type. We investigated the relationship between learning and the bold-shy behavioral type by comparing performance of bold and shy zebrafish in conditioned place preference (CPP) and 2-choice tasks. Bold zebrafish learned significantly faster than the shy fish but there were no differences in their final performance in the CPP. When tested in the 2-choice task, we found no clear evidence of learning, however bold fish made more initial choices than shy fish. Overall, our study suggests that bold zebrafish tend to be faster learners when compared to shy zebrafish. The lack of differences in the final change in behavior suggests that learning differences may be due to how the animals initially interact with stimuli because of differences in neophobia.

**Supplementary Information:**

The online version contains supplementary material available at 10.1038/s41598-025-00423-6.

## Introduction

As animals interact with their environment, how quickly they learn and recall these interactions can vary between individuals^7,11^. It has been hypothesized that variation in learning between individuals can be partly explained by differing personality types^16,22,48,49^. Across many animal taxa, studies demonstrate that one common dimension of personality is the bold-shy continuum^44^. Bold individuals are characterized by displaying lower neophobic and stress-related behaviors and have higher exploratory activity. In contrast, shy individuals tend to have opposing traits^2,48,56^.

However, studies across taxa find a conflicting relationship between personality and learning. Many studies showed that bold individuals learn faster than shy, in animals such as mammals, birds, and teleost fish^5,13,15,19,23,26,36^. Fewer studies found the opposite (e.g. shy learn faster than bold) or no relationship between personality and learning speed^3,32,37,50^. Inconsistencies across studies suggest that the relationship between learning and personality may be complex and depend on other factors of the task.

Individuals of differing personality types vary in their interactions with different learning tasks or stimuli, which may influence learning performance^49^. For example, different training paradigms require that the animal engage with the stimulus in different ways. Some studies found that learning is not correlated across training paradigms^12,18,24,27,40^. Similarly, a meta-analysis in non-human animals found a low correlation between learning ability across cognitive tasks^40^. This potential variation across tasks suggests a need for measurements in multiple learning tasks^20^. One specific way that tasks can vary is how an association is formed (e.g., operant versus classical conditioning). During operant conditioning an animal must perform an action before receiving a stimulus whereas in classical conditioning no prior active movement by the animal is required to form an association. Prior studies have demonstrated that between classical and operant tasks, both learning performance and underlying physiological mechanisms differ^8,9,34^. Currently there are no studies investigating how the relationship between personality and learning varies across classical and operant tasks. Of note, neophobia, which is elevated in those with a shy personality type, has been seen to affect operant learning of a food reward due to higher latencies to approach^52^. Thus, comparing a passive (e.g., classical conditioning) task that does not require the animal to approach a novel object to an active (e.g., operant conditioning) task that does require an approach may cause the relationship between personality and learning to vary.

Investigations of effects of personality type on cognition (e.g., learning) have mainly relied on investigators conducting behavioral screens of a population to obtain bold and shy personality types^5,13,15,19,23,26,32,36,37,50^. However, studies across multiple species have successfully artificially selected for personality type^39,41,54,55^. Wong et al., (2012) created two lines of zebrafish that were selectively bred to display opposing exploratory behavior in response to a novelty stressor (HSB and LSB lines)^60^. Zebrafish of these lines consistently differ in their behavior across both contexts and time^2,25,60^. More specifically, across six different stress and anxiety behavioral assays, the HSB line exhibits a greater amount of stress and anxiety-like behaviors (e.g. high freezing, less exploration, high neophobia) than the LSB line^25,60^. The exploratory behavior of the lines in an open field test is repeatable and reliable across time^2^. Further, the HSB line releases cortisol faster under stress compared to the LSB line^58^. The lines also differ in basal neurotranscriptome profiles^59^, neuromolecular responses to anxiolytic drugs^20,57^, and contextual fear learning^3^ but not gut microbiota^1^. Taken together, use of selective breeding to reliably produce individuals that show the bold (LSB) or shy (HSB) personality types can facilitate studies investigating learning processes.

**Fig. 1:**
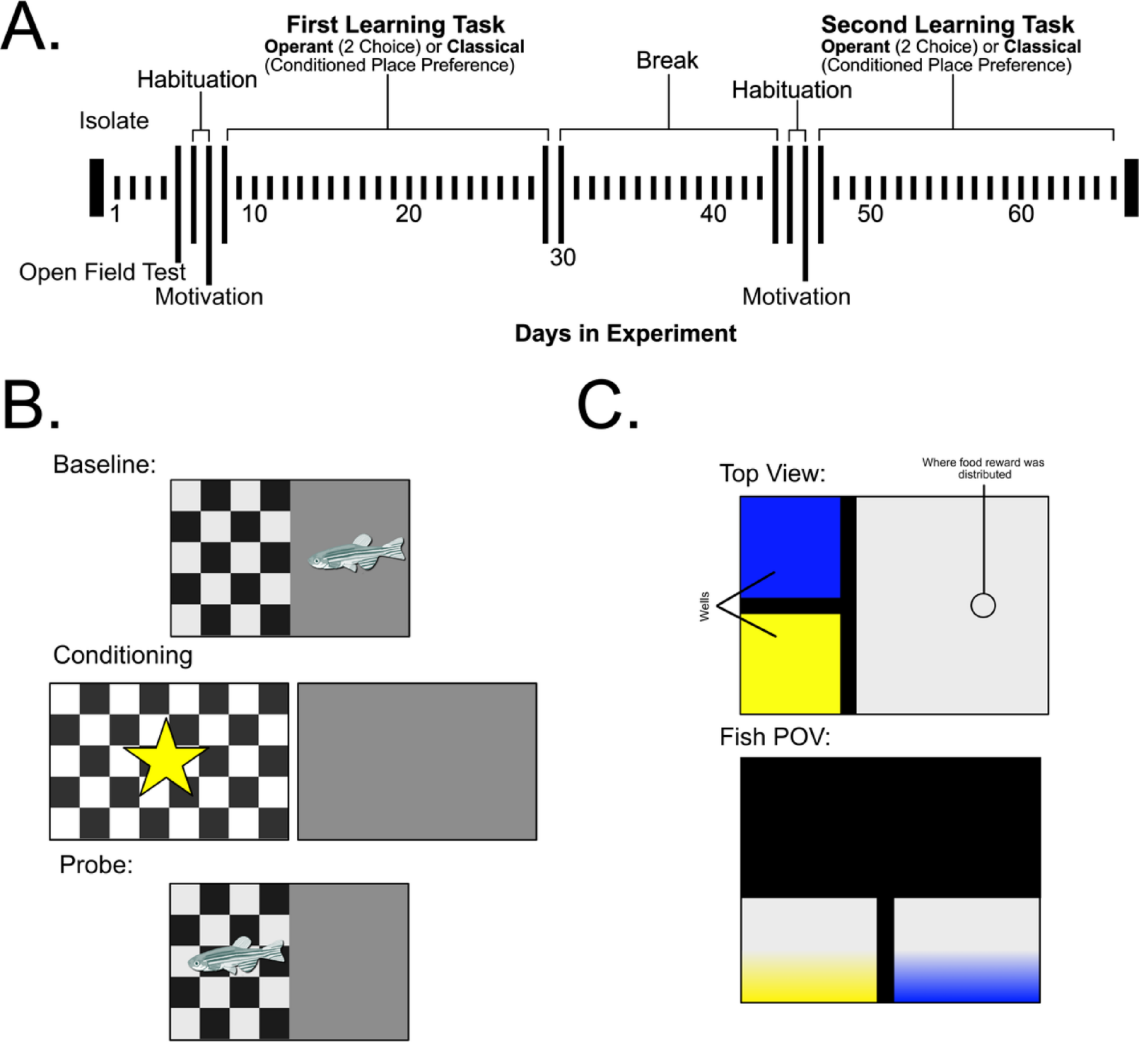
Overview of Experiment Timeline and Tasks. (**A**) Top view and the fish point of view of the 2 choice discrimination task. The wells and where the food reward was distributed are labelled. (**B**) Brief overview of the stimulus displayed to the fish during the conditioned place preference task. The fish are initially presented the check and the grey stimulus. Whichever is least preferred is rewarded in conditioning trials, shown by the star symbol. Then the probe presents both stimuli to the fish and the preference is measured again. (**C**) Fish of all groups started with isolation on the first day and then went through an open field test (OFT), habituation, motivation test, and training on the first task (either conditioned place preference or 2-choice discrimination task). After a break fish went through habituation, motivation test, and the second task (whichever task was not completed first). The study design was counterbalanced where half of the fish began with the CPP task while the other half began with the 2-choice discrimination task.

In this study, we investigated the effect of personality type on learning performance across two associative learning paradigms in zebrafish (*Danio rerio*). Additionally, we conducted an open field test and a motivation test to confirm personality type and assess motivation for the food reward, respectively. Using a within-subjects and counter-balanced design we individually trained bold (LSB) and shy (HSB) zebrafish to associate a visual stimulus with a food reward in both conditioned place preference and 2-choice tasks. The conditioned place preference task was a classical task in which the animal was trained to associate a color/pattern with a food reward (Fig. 1b). In the 2-choice task the fish had to swim into a specific colored area to receive the reward (Fig. 1c). Through use of these tasks, one goal was to assess for consistency of learning performance in each personality type across distinct reinforcement requirements. We tested the prediction that bold individuals will be faster learners compared to shy fish because of their decreased neophobia. We also evaluated the prediction that there will be an interaction effect of personality and training paradigm on learning speed. Given that the operant task requires fish to actively make a choice, we expected that there would be a larger difference between bold and shy fish compared to the classical task due to the increased neophobia in shy fish.

## Methods

### Animals

We used zebrafish from lines that were selectively bred in the lab from wild caught zebrafish, near the village of Gaighata, India (approximately 60 km from Kolkata) from a commercial supplier (Dolphin International, Los Angeles, CA, USA) in 2005, to exhibit a shy (HSB) or bold (LSB) personality type (*n* = 48 per line)^60^. The fish used in this study were selectively bred for 13 generations. Before testing, we housed the fish together in 40 L tanks and fish were fed twice a day with Tetramin Tropical Flakes (Tetra, USA). One week prior to testing we physically isolated fish into 3-liter tanks on a recirculating water system (Pentair Aquatic Eco-Systems or Aquaneering) using UV and solid filtration on a 14:10 L/D cycle, with lights on between 06:00–20:00 and a temperature of 27 °C. Fish had visual and olfactory access to each other and remained in this environment when not being tested for the duration of the experiment. Starting three days before testing we withheld food from the fish to reduce the possibility of satiation while training. We determined the sex of each fish using visual inspection of sexually dimorphic characteristics^29^.

## Behavioral assays overview

We conducted four behavioral assays on each fish: an open field test (OFT), a test for food motivation, a conditioned place preference (CPP, classical conditioning) task, and a 2-choice discrimination task (operant conditioning). The OFT and food motivation tests were performed before either learning paradigm. Using a within-subjects design, we tested each fish in both associative learning paradigms and counterbalanced the starting paradigm (Fig. 1). We used frozen adult brine shrimp (*Artemia* spp., San Francisco Bay Brand, USA) administered in liquid form as the food reward for the treatment fish. Half of the fish served as controls and received distilled water instead of brine shrimp. We started with four groups of 24 fish (bold control, shy control, bold treatment, and shy treatment). A power analysis revealed that 12 and 22 fish per group for the CPP and 2-choice task, respectively, were required to achieve 80% power. We used an estimate of medium effect size for the CPP and the pilot data to determine the r squared for the 2-choice task. Therefore, we had 24 fish per group to allow for unpredictable events during the experimental process (e.g., fish mortality, equipment malfunction). All behavioral assays were performed between 3 and 8 h after light onset. After 4 days of isolation, we tested each fish in the open field test to confirm behavioral phenotype differences between the lines. We then habituated each fish for two consecutive days in the conditioning tank. We assessed biases in food motivation for the brine shrimp before starting baseline trials of the associative learning assays on day 7. On day 8 we started training in one of the learning paradigms. Each fish had a 14 day inter-assay testing interval to minimize the influence of the tasks on each other. During testing in learning paradigms, treatment fish were only fed during the conditioning and control fish were fed brine shrimp in their housing tank.

## Open field test

We individually tested fish in an OFT in a tank that was 31.75 cm x 31.75 cm x 10 cm containing 4 L of water following established protocols^2,50^. We placed the fish in the tank and video-recorded its behavior for 5 min. We used Ethovision XT 17 (Noldus, Netherlands) to quantify the amount of time that each individual spent frozen during the trial. Time frozen was defined as the duration of time with linear movement slower than 0.5 cm/sec.

## Motivation test

This test was performed in the AD and LT models of Zantiks semi-automated behavioral units (Zantiks, Cambridge, UK). After acclimating for 30 s, a food reward was administered 3 times at 30 s intervals. We quantified the time spent in a 9 × 12 cm rectangle centered around the food administration tube. The behavior was recorded starting immediately after the first brine shrimp administration until the end of the test to measure the motivation of the fish for the food reward. We performed the motivation test in both Zantiks models but due to the size and height of the tank in the larger LT unit, the food drifted outside the rectangular fish tracking zone. Thus, we only used the data from the AD unit to assess motivation in this motivation test.

## Conditioned place preference

We used a modified conditioned place preference protocol^30^ in the Zantiks LT unit. The testing tank (36 cm x 27 cm x 30 cm) was filled with 5.8 L of water. We tested each fish in the CPP task for three weeks which consisted of 2 days of habituation, 1 day of baseline testing, 11 days of conditioning, and 3 days of probe trials. To habituate each fish to the assay we placed the fish in the tank for 10 min with no training stimulus lights. After habituation, we determined the baseline preference for the light stimuli (grey or checkered pattern) for each fish. Fish swam freely for 10 min in the tank where one half was illuminated from the bottom with a grey screen and the other half a checkered screen. We determined the conditioned and non-conditioned stimuli as the stimuli where the fish spent the least and most amount of time, respectively. During conditioning days, we sequentially presented each stimulus for 5 min to each fish. The non-conditioned stimulus was presented for the first five minutes followed by the conditioned stimulus. One hundred microliters of brine shrimp or distilled water were administered every minute during the presentation of the conditioning and non-conditioning stimulus, respectively. Food reward consisted of 11.4 g of frozen brine shrimp in 30 mL of distilled water. We fed control fish an equivalent amount of brine shrimp after each conditioning trial. Probe trials were administered the day after a conditioning trial. Probe trials were conducted 3, 7, and 11 days after the first day of conditioning with a total of 3 probe trials. Probe trial methods were the same as those used in the baseline preference step where we quantified the time spent in each stimulus for each fish. The order of stimulus presentation was consistent within a fish but random across fish for probe and baseline trials. We compared the time spent in the conditioned zone at baseline to the time spent in the conditioned zone in probe trials to assess learning.

### 2-choice discrimination task

We used a modified 2-choice discrimination task from an established protocol^6^. We used the AD model Zantiks unit (Zantiks, Cambridge, UK) with a 14 cm x 20 cm x 15 cm tank filled with 2.5 L of water. We habituated each fish for 20 min a day for two consecutive days with white lights on in the wells (where the light stimulus was presented). We tested each fish every other day for a total of 10 testing days. Fish were fasted on non-testing days. In this task, the fish were presented with two 6.5 cm x 5.1 cm light stimuli (blue and yellow) from below at one end of the tank. Prior studies show that with appetitive learning in zebrafish, there is a bias towards red compared to other colors such as blue and yellow^28,51^. For each fish, a color was randomly chosen at the start of testing to be the reinforced stimulus where a food reward (brine shrimp) was administered at the other end of the tank when the fish swam into the designated reinforced color. The food reward consisted of 5.7 g of frozen brine shrimp suspended in 30 mL of distilled water. Each trial began with an acclimation period of two minutes with white lights in the two wells. After two minutes blue and yellow lights were presented for 30 s. Swimming into the designated correct choice resulted in the correct colored light staying on for an additional 30 s and we simultaneously administered 25 µl of the food reward. An incorrect choice resulted in all lights turning off for 30 s. This sequence ran for a total of 20 trials each day for each fish (i.e., one session consists of 20 trials). The position of the yellow and blue lights (e.g., left or right) was randomly set for each trial. There was an intertrial interval of 10 s. Control fish underwent the same protocol with distilled water administered instead of brine shrimp and were fed brine shrimp after each testing day. We compared the number of correct choices and the total number of choices across sessions to assess learning. We initially piloted this task using above procedures with a domesticated zebrafish strain (*N* = 12 per group, purchased from a local pet store, PetSmart) and found a significant effect of treatment and day on correct choices, suggesting that the fish were learning (Table S7).

### Statistical analysis

We performed all statistical tests using R statistical software (R 4.2.2 GUI 1.79 Big Sur ARM build) and Rstudio version 2022.12.0 + 353^42,47^. Due to fish mortality during the experiment, the final sample sizes for statistical analyses between the conditioned place preference (bold control (*n* = 20), shy control (*n* = 20), bold treatment (*n* = 19), and shy treatment (*n* = 19)) and 2-choice (bold control (*n* = 20), shy control (*n* = 17), bold treatment (*n* = 19), and shy treatment (*n* = 20)) tasks differed. We conducted post-hoc tests using the emmeans^31^ package and normality and assumptions were checked using base R. The lme4 package^4^ was used to test negative binomial linear mixed effect models. We obtained simple statistics for all measures using the psych package^45^ (Table 1). Model assumptions, including normality were inspected in R.

To investigate differences between bold and shy groups in the open field test and motivation test, we used a Welch two-sample t-test. We compared the duration of time frozen in the OFT between the bold and shy personality types. To investigate differences in food motivation, we compared the duration of time spent around the food administration tube between the bold and shy personality types.

In the conditioned place preference task, we modeled the duration of time spent in the conditioned stimulus for the last half (5 min) of the baseline and probe trials to test for a change in preference for the conditioned stimulus across the task within the different groups. We did not include the first half (5 min) in the analysis to minimize the influence of handling on fish behavior. We performed a repeated measures ANOVA to investigate the effects of treatment, sex, personality type, and conditioning day on the time spent in the conditioned stimulus. We included the main effects of treatment, personality, sex, and conditioning day as well as all interactions and used type III sums of squares. We used a White correction because there was some heteroskedasticity between personality type. We used Tukey post-hoc tests to evaluate differences in the response variables across trials for each group and within trials between groups. To further test the relationship between neophobia and the speed of learning we performed a Pearson’s correlation between time spent frozen in the OFT and the change in time spent in the conditioned zone from baseline to after 3 days of conditioning for control and treatment fish separately. We also tested for a relationship between learning speed and final learning performance through a Pearson’s correlation between change in CS time from baseline after 3 days of conditioning and final time spent in the conditioned stimulus for the bold and shy fish.

We modeled the number of correct choices over the conditioning days to examine changes in correct choices over time within groups for the 2-choice discrimination task. We performed a negative binomial mixed effect regression on the number of correct choices with treatment, personality type, and session as the fixed effects and ID as the random effect. Simple slopes were obtained to test for increases in correct choices within each group using the interactions package in R and plotted using the same package^33^. Additionally, after noticing a difference between bold and shy fish, we performed a negative binomial mixed effect regression on the total number of choices with treatment, personality type, and session as the fixed effects and ID as the random effect. We also obtained simple slopes for this model. We initially included sex as a factor in both models for the 2-choice discrimination task analyses but did not observe any significant main effects of sex or interactions involving sex. We therefore removed sex from the models for the 2-choice discrimination task analyses.

## Results

### Shy fish freeze more but have equal motivation to eat

**Fig. 2:**
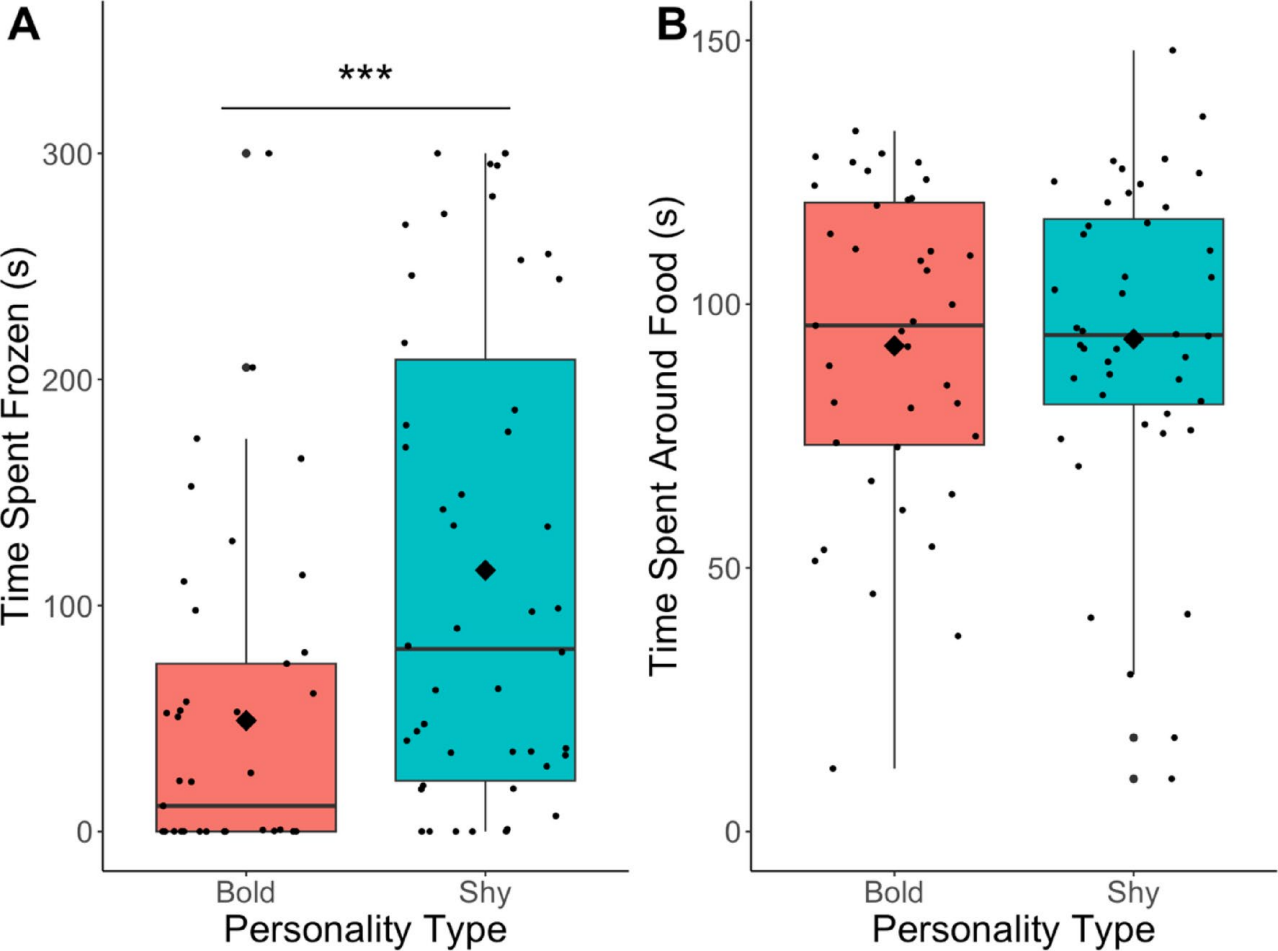
(**A**) Boxplot of time spent frozen in the open field test. (**B**) Boxplot of time spent around the food in the motivation task. Bold fish are in red and shy fish are in teal. The diamond indicates the mean and the line is at the median. ***, *p* < .001.

There was a significant effect of personality type on freezing time in the open field test (Fig. 2a). Shy fish spent significantly more time frozen than bold fish (t =-3.55, df = 90, *p* = 6.4*10^− 4^). There were no significant differences between personality types (t = -0.19, df = 82, *p* = .85) in the amount of time spent around the food in the motivation task (Fig. 2b).

### Bold male fish change their behavior before shy female fish

For the full model of the effects of sex, treatment, personality, conditioning day and all interactions on the time spent in the conditioned zone there was no main effect of sex (F(1,210) = 0.64, *p* = .43), but there were significant effects of treatment (F(1,210) = 8.25, *p* = .005, η² = 0.006), personality (F(1,210) = 4.47, *p* = .04, η² = 0.04), and conditioning day (F(3,210) = 7.57, *p* = 1.0*10^− 4^, η² =0.037), (Table S1). There was also a significant interaction between treatment and conditioning day (F(3,210) = 8.43, *p* = 3.7*10^− 5^, η² = 0.04) and between personality, treatment, sex and conditioning day on the time spent in the conditioned zone (F(3,210) = 3.67, *p* = .02, η² = 0.018).

Tukey’s post hoc tests of the personality, treatment, sex and conditioning day interaction effect on time spent in the conditioned zone at various time points revealed that there was a significant increase from baseline in bold treatment males after 3 (t = -3.49, df = 210, *p* = .003), 7 ( t = -5.24, df = 222, *p* = .0001), and 11 (t = -4.66, df = 210, *p* = 0001) conditioning days. For shy treatment females, there was a significant increase from baseline after 7 (t = -3.23, df = 210, *p* = .01), and 1 (t = -3.23, df = 210, *p* = .01) conditioning days (Table S2). No other groups had significant differences from baseline (*p* > .05, Table S2).

There was no significant correlation between learning speed (change in CS time from baseline after 3 days of conditioning) and final time spent in the conditioned stimulus in the CPP task for the bold fish (ρ = 0.19, *p* = .44) or the shy fish (ρ = 0.22, *p* = .35). There was a significant negative correlation (*r* = -.43, *p* = .0065) between time spent frozen, and the change from baseline to after 3 days of conditioning for treatment fish. There was no significant correlation between time spent frozen and the change from baseline to after 3 days of conditioning for control fish (*r* = -.13, *p =* .43).

### No evidence of learning in 2-choice discrimination task with correct choices

In the 2-choice discrimination task there was no significant difference in the number of correct choices between control and treatment fish (Table S3). There was only a significant main effect of personality type such that bold fish made more correct choices compared to shy fish (b = − 0.49, t = -2.84, *p* = .01) and a significant interaction between personality type and session (b = 0.03, t = 2.601, *p* = .01). Testing for the simple slopes (Table S4, Fig. 4a), both shy control (m = 0.03, t = 3.15, *p* = 2.2*10^− 5^) and shy treatment (m = 0.04, t = 4.24 *p* = 4.4*10^− 6^) groups had a significant positive slope while bold control (m = 0, t = -0.26, *p* = .79) and bold treatment (m = 0.01, t = 0.98, *p* = .33) have no significant slopes.

**Fig. 3:**
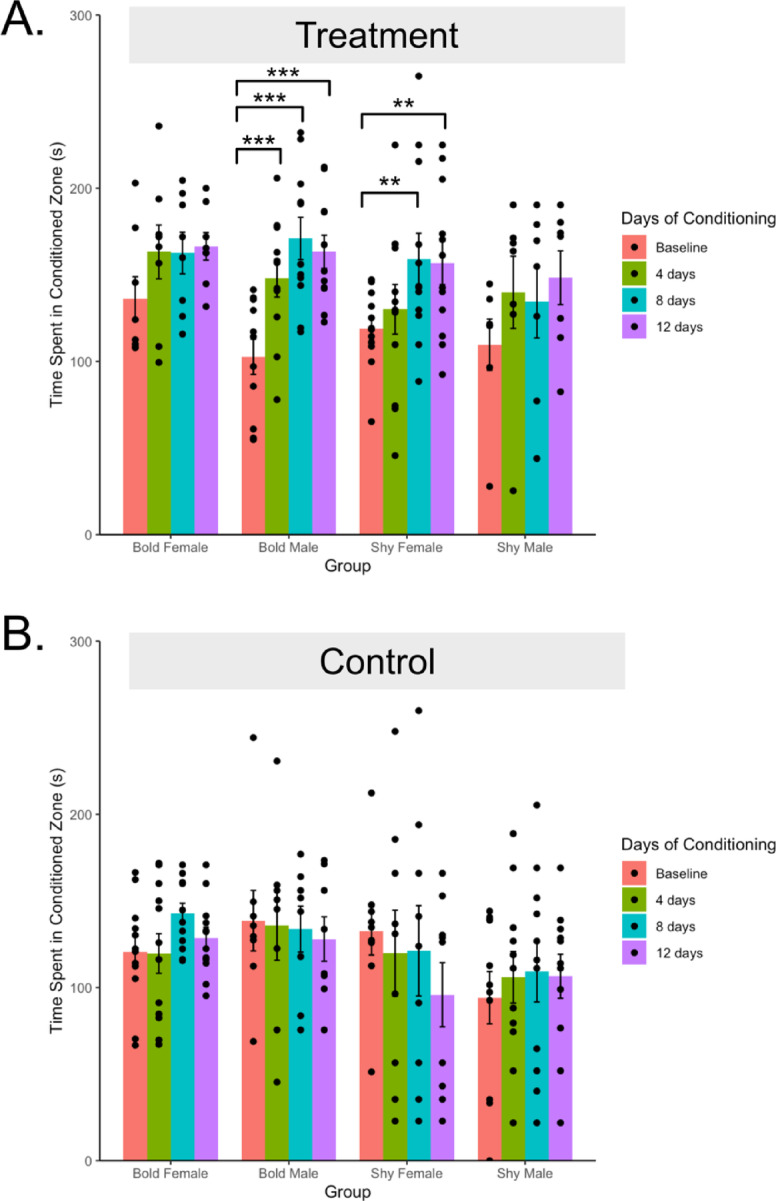
Time spent in the conditioned zone by group and day of conditioning in the CPP. (**A**) shows treatment fish and (**B**) shows control fish. Red bars are at baseline, blue bars are after 3 days of conditioning (Probe 1), green bars are after 7 days (Probe 2) and purple bars are after 11 days of conditioning (Probe 3). Error bars indicate standard error. *p* < .1, *p < .05, **p < .01, *** *p* < .001.

*Difference across treatment and control only in the total number of choices in 2-choice discrimination task*.

**Fig. 4:**
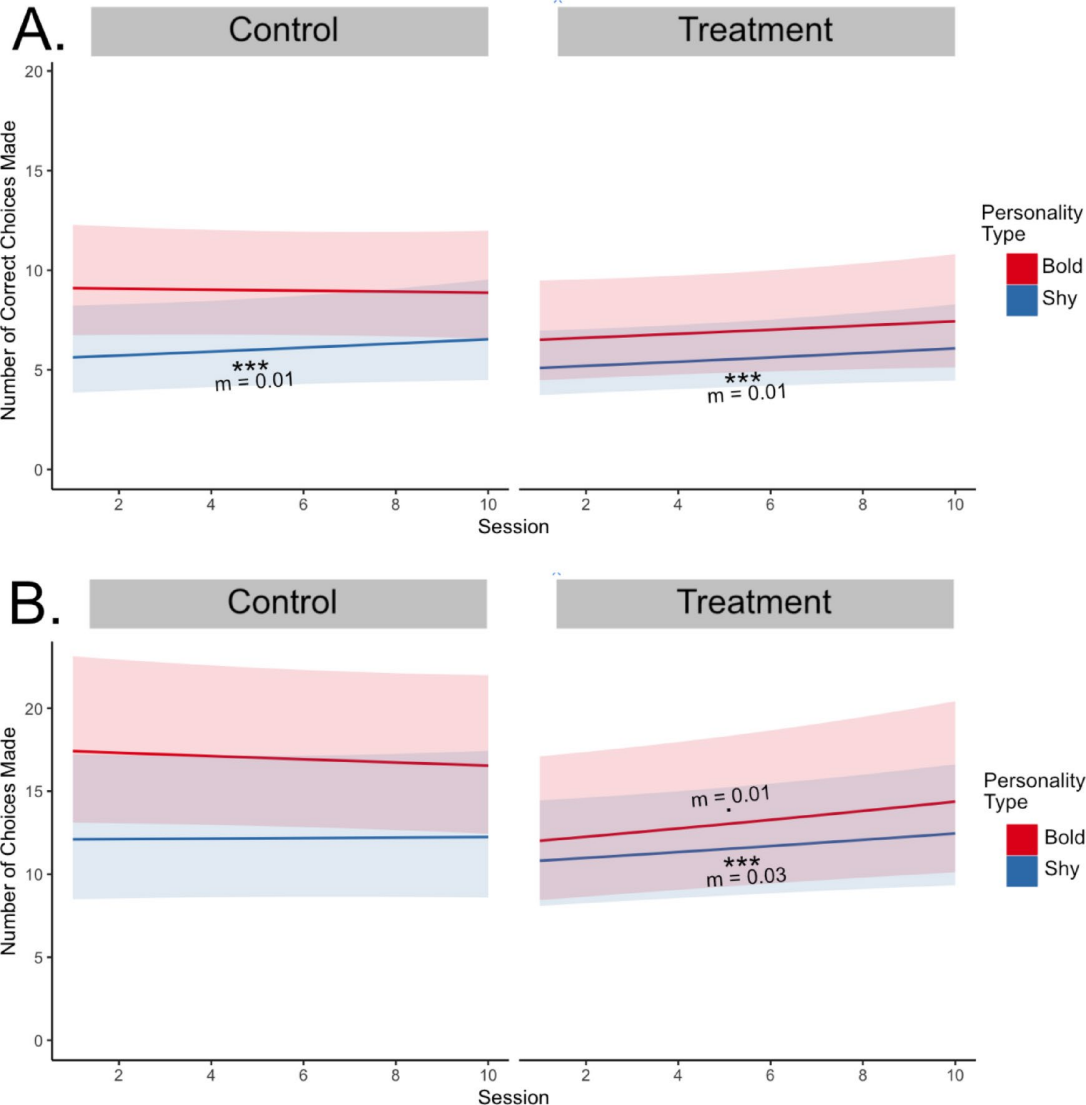
Regression lines of the number of choices made and number of correct choices made by personality type and treatment. (**A**) shows the regression lines for control and treatment fish for the number of correct choices made (**B**) shows regression lines for control and treatment fish for the number of choices made. The bold group is in red and the shy group is in blue. Shaded regions indicate a 95% confidence interval. ., *p* < .1; ***, *p* < .001.

For the total number of choices, there was a significant difference between control and treatment fish (Fig. 4b, Table S5). There was a main effect of personality type on total number of choices (b = − 0.34, t = -2.07, *p* = .04) where bold fish had a higher total number of choices than shy. There was a trend for an interaction effect between session and treatment (b = 0.15, t = 1.69, *p* = .09). Testing for the simple slopes, shy control (m = 0.01, t = 1.22, *p* = .22), and bold control (m = 0, t = -0.44, *p* = .66) did not have a significant relationship (Fig. 4a). Only shy treatment (m = 0.03, t = 4.59, *p* = .4.4*10 − 6) had a significant positive slope (Fig. 4b, Table S6). In contrast, bold treatment had a slope approaching significance (m = 0.01, t = 1.90, *p* = .06) (Fig. 4b).

## Discussion

Variation in learning performance can be due to complex interactions between intrinsic (e.g., personality type) and extrinsic (e.g. learning task) factors^49^. We investigated the effects of personality types and learning task by testing zebrafish of differing personalities across two different associative learning assays. Overall, we found that learning performance in one of the tasks was influenced by an animal’s personality type.

Bold and shy fish differed in learning speed in the conditioned place preference task in a sex dependent way. We observed that for treatment groups bold males and shy females showed evidence of learning while bold females and shy males did not (Fig. 3a). A study in great tits found a similar sex dependent relationship for behavioral flexibility^53^. In wild zebrafish the effect of sex on personality and cognition varied by geographic location^14^. A meta-analysis of the effect of personality on cognition found that the relationship between personality and learning was male specific^11^. Collectively, this suggests that environmental or genetic factors may change the effect of sex on personality and cognition. While the environmental and genetic factors that caused this effect in our fish are unknown, this result points to the importance of investigating sex in the relationship between personality and learning. We also cannot rule out that due to smaller sample sizes for the bold females and shy males (*N* = 8 and *N* = 7, respectively), there was not enough power to detect differences in these groups given the suggested group size of 12.

Treatment bold fish increased the time spent in the conditioned stimulus earlier than treatment shy fish in the conditioned place preference task, which suggests that the bold fish learned faster in this task. This effect is primarily driven by performances of treatment bold males and shy females. Treatment bold males showed a significant increase in time spent in the conditioned stimulus after just 3 conditioning days whereas shy female fish did not show an increase from baseline until 8 days of conditioning (Fig. 3a). As expected, control groups showed no evidence of learning regardless of personality type or sex (Fig. 3b). Due to probe tests occurring after several days of conditioning, we cannot rule out that the fish could have learned (Fig. 3). These results are consistent with other studies demonstrating that individuals with bold personality types learn faster than shy individuals^5,13,14,19,23,26,36^. Differences in learning speed between personality types in this task may be due to behavioral differences in stress reactivity, exploration, and neophobia^49,50^. One behavior that does not explain the difference is motivation as there were no significant differences in motivation for the food stimulus between personality type (Fig. 2). Interestingly, there were no differences in the amount of time spent in the conditioned stimulus between the personality types after 11 days of conditioning, suggesting that individuals approach an asymptotic level of performance. This suggests that both personality types are capable of changing their behavior (e.g. learning) to similar extents and therefore differences in cognitive ability between personality types is an unlikely explanation for differences in learning speed.

When testing the same fish in the 2-choice discrimination assay, there was no significant difference in the number of correct choices between treatment and control groups, which suggests the fish did not learn the stimulus-reward association in this task (Fig. 4). However, there were differences across personality types in which both shy treatment and control increased the number of correct choices while the bold groups did not. The positive slope for the shy groups is likely due to an overall increase in total choices with repeated exposure. When looking at the total number of choices made over sessions, the control groups did not change over time while the treatment groups increased the total number of choices made over sessions. This suggests that the treatment fish did not learn the color association but may instead have learned to go into the wells where the food was administered regardless of color. Animals can attend to several cues in discrimination learning and sometimes attend to unintentional or general cues^35^. We also cannot rule out that rewarding the fish in a different location than the stimuli may have decreased the strength of pairing between action and reward^38^. While a pilot study using the above 2-choice paradigm resulted in a significant increase in number of correct choices for a domesticated strain of zebrafish (Petsmart) (Table S7), we cannot rule out that additional training sessions are needed for the bold and shy zebrafish strains used in this study due to possible strain differences. As the fish in the current study did not show evidence of learning in the 2-choice task, we were unable to directly compare performances between the CPP and 2-choice tasks. Despite no evidence of color association learning in the 2-choice discrimination task, the bold fish made more choices than shy fish in the first session. This is likely due to decreased neophobia and increased exploration in the bold fish as demonstrated in this strain and other species^48,60^.

Differences in neophobia (e.g. latency to approach novel objects) classically distinguish bold and shy personality types^10,48,56^. In the current study, one potential explanation for bold fish learning quicker in the conditioned place preference and making more initial choices in the 2-choice discrimination task relative to shy fish are differences in neophobia between the personality types. The shy fish could have found the colored lights in the 2-choice discrimination task initially aversive and increased their choices as they habituated to the novel stimuli. Shy individuals tend to have increased neophobia and habituate slower, which would result in the shy fish taking longer to make active choices^10^. The two days of habituation in the 2-choice discrimination task allowed the fish to experience the tank and lighted wells but at the start of conditioning, they were naïve to the color of the lights and the changing stimuli. A similar effect was seen in *Gallus gallus* where less exploratory individuals (i.e., shy) habituated slower to a loud sound than those that were more exploratory^17^. Neophobia may also explain shy fish learning slower in the CPP task. Shy fish could have experienced more stress than the bold fish at the start of the task even after habituation and so learned the positive association slower. In this study, we tested freezing behavior in response to a novel environment through the open field test. While not classically viewed as a neophobia paradigm, the open field test can be used as a measure of fear of a novel space^21^. Fish that showed higher freezing duration in the open field test, which is positively associated with other neophobic behaviors in these selectively bred zebrafish lines^25,60^, had slower changes in conditioned behavior. Supporting the effect of neophobia on learning performance, mollies (*Poecilia mexicana*) that were desensitized to the lights and sounds used in the task showed no differences in learning related to personality type^50^. Increasing familiarity with the task environment and stimuli could explain why shy fish were slower to increase their preference for the conditioned stimulus but ultimately reached a level of performance similar to bold fish after 11 days of conditioning. Bold individuals tend to make associations faster likely because they are less neophobic and in a simple conditioned place preference task, this leads to them learning faster but does not change the plateau of performance^13,15,19^.

The relationship between faster learning and bold personality type is not consistent across all studies^32,37^. Potential explanations are that the relationship between speed of learning and personality can depend on aspects of the task such as learning stimulus valence or task complexity. Shy zebrafish trained in a contextual fear learning paradigm showed faster learning than bold zebrafish^3^. As shy zebrafish have a faster glucocorticoid response to a novelty stressor than bold fish, this may facilitate quicker learning of aversive stimuli^43,46,58^ but inhibit learning of appetitive stimuli observed in the current study. Task complexity may also have an effect as a study looking at learning accuracy found that aggressive spiders (e.g. bold personality type) were more accurate in a simple task but not in a more complex task^12^. Future work may consider testing whether the same trend holds in a more complex classical conditioning task. In a more complex task, bold fish may make incorrect associations and not learn as quickly as shy fish.

Overall, we found support for differences between bold and shy zebrafish in how they interact with two different learning tasks. In a 2-choice task requiring an active behavioral response, we found differences in the initial number of choices made between personality types, suggesting that the personality types naively interacted with the stimulus differently. In the conditioned place preference task, the bold fish learned faster than the shy fish but both achieved similar levels of performance. In this task, we also found sex-dependent differences such that bold males and shy females learned faster. Differences in performance between bold and shy individuals in both tasks may be explained by variations in neophobia related to personality type.

## Electronic supplementary material

Below is the link to the electronic supplementary material.

Supplementary Material 1

## Data Availability

All the data that was used for this project is attached in the supplemental information. Table S8 shows the data from the OFT and Motivation tasks. Table S9 shows the data from the conditioned place preference task and table S10 shows the data from the 2-choice task.
