# Supplementary material for: Bold zebrafish (Danio rerio) learn faster in a classical associative learning task
Source: Sci Rep. 2025 May 10;15:16331. doi: 10.1038/s41598-025-00423-6 (PMC12065844; doi:10.1038/s41598-025-00423-6)
Supplement: Supplementary file 1 — Supplementary Material 1 [file 41598_2025_423_MOESM1_ESM.docx]

**Supplementary Tables and Figures**

Bold zebrafish (*Danio rerio*) learn faster in an associative learning task

Scientific Reports

Jamie Corcoran^1^

Levi Storks^2,3^

Ryan Y. Wong^1,2^

^1^University of Nebraska at Omaha Psychology Department, Omaha, NE USA

[Jcorcoran@unomaha.edu](mailto:Jcorcoran@unomaha.edu) ORCID: <https://orcid.org/0009-0001-7880-475X>

[rwong@unomaha.edu](mailto:rwong@unomaha.edu) ORCID: https://orcid.org/0000-0003-0236-672X

^2^University of Nebraska at Omaha Biology Department, Omaha, NE USA

^3^University of Detroit Mercy Biology Department, Detroit, MI US

**Supplementary Table 1**

*ANOVA of treatment, personality type, sex, and probe on the amount of time spent in the conditioned zone in the conditioned place preference task*

*
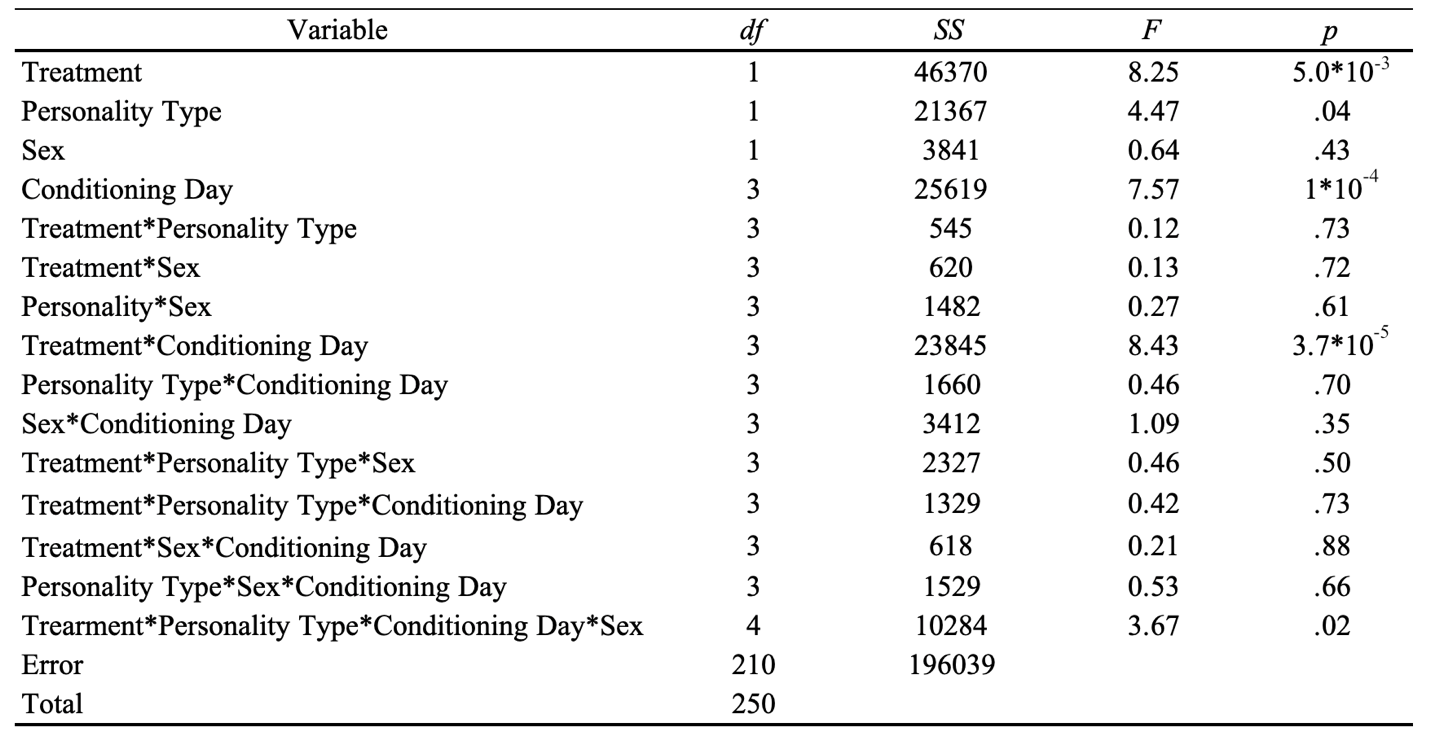
*

**Supplementary Table 2**

*Tukey post hoc comparing time spent in the conditioned zone at baseline, probe 1 (3 days of conditioning), probe 2 (7 days), and probe 3 (11 days) in the conditioned place preference task between personality and sex*

**
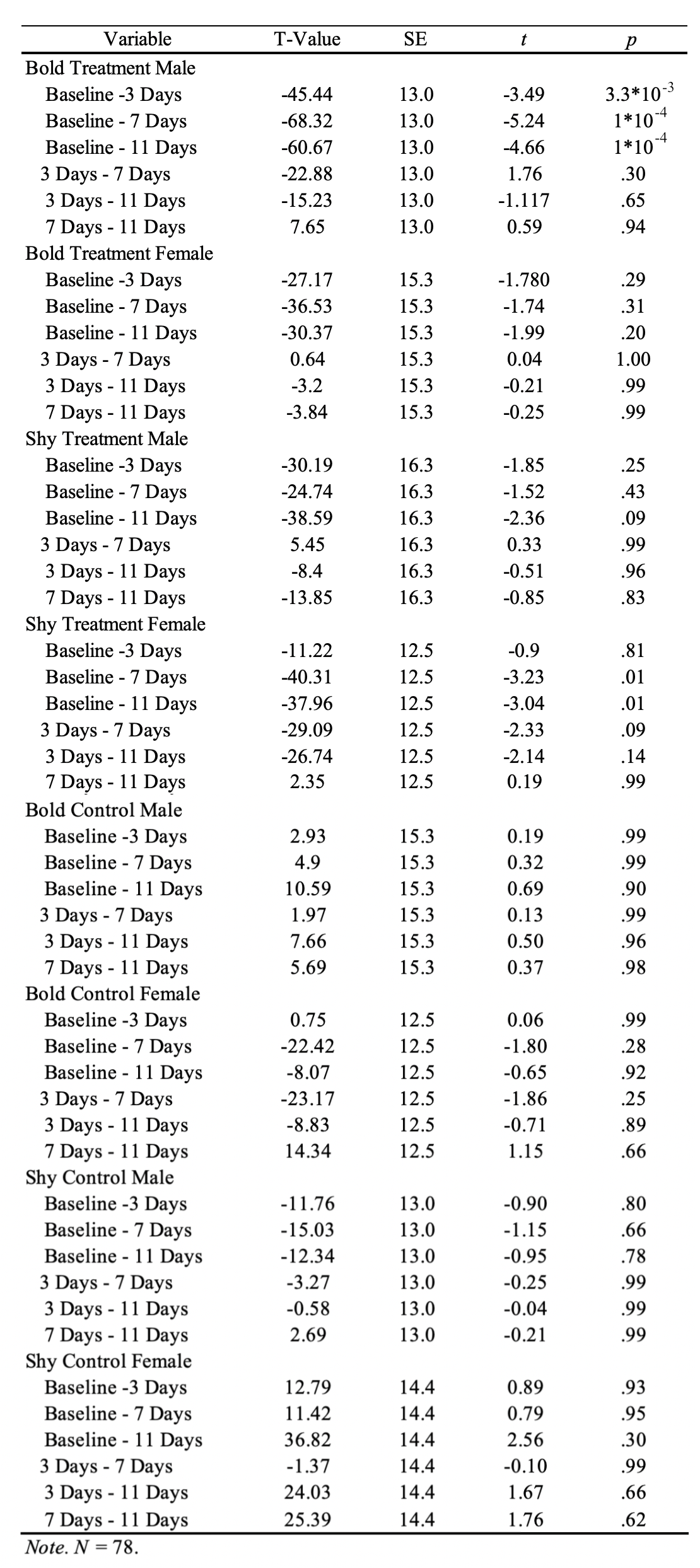
**

**Supplementary Table 3**

*Negative binomial mixed effect model of session, treatment, and personality type on the number of correct choices made in the 2-choice discrimination task*

*
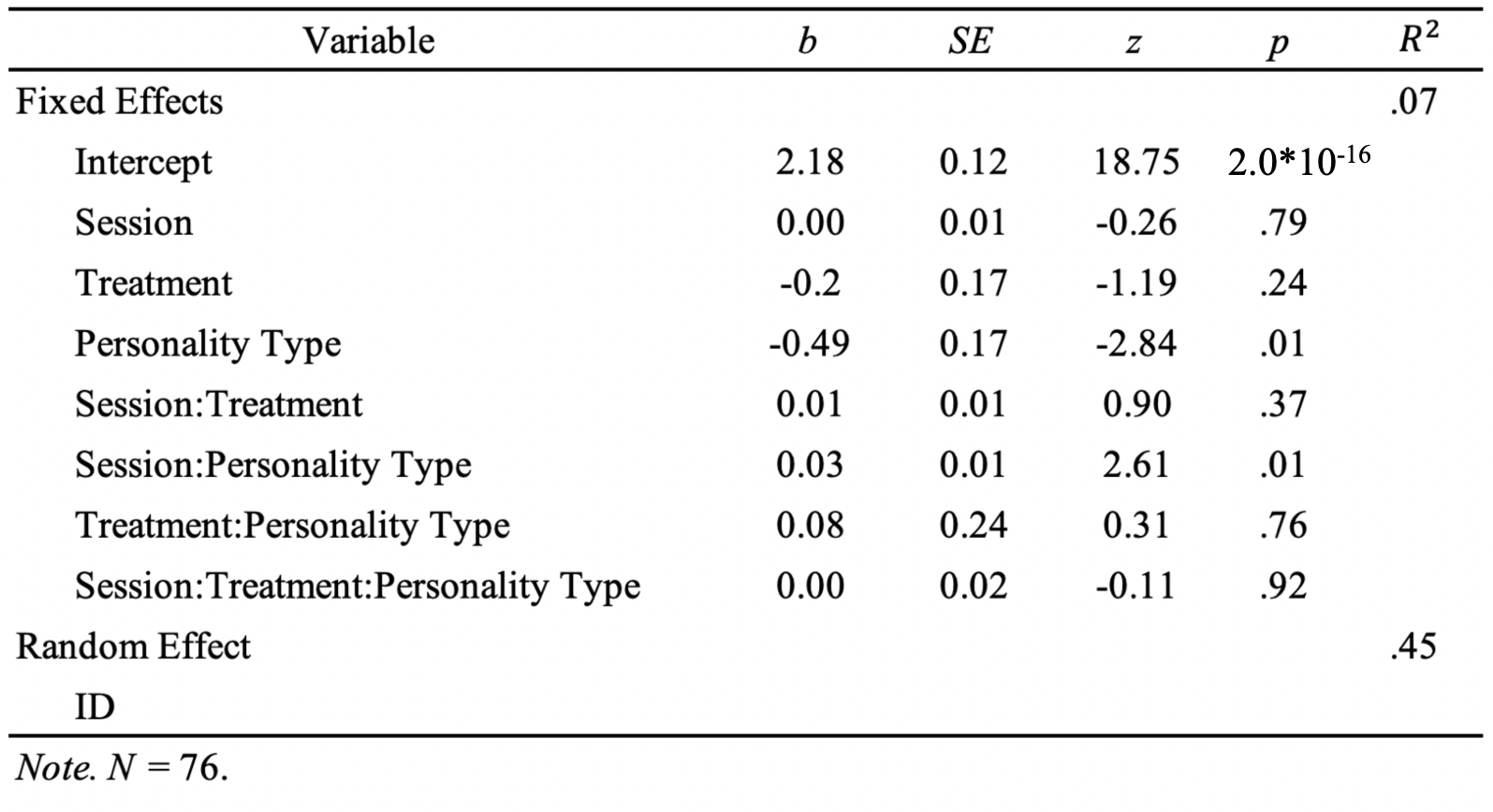
*

**Supplementary Table 4**

*Simple slopes of negative binomial mixed effect model of session, treatment, and personality type on the number of correct choices made in the 2-choice discrimination task
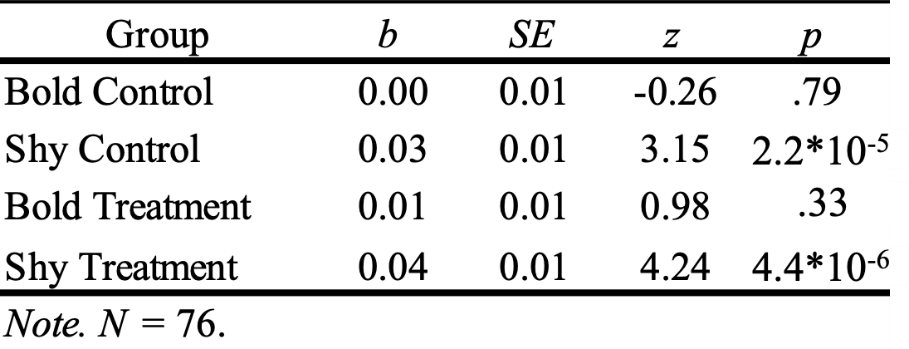
*

**Supplementary Table 5**

*Negative binomial mixed effect model of session, treatment, and personality type on the number of choices made in the 2-choice discrimination task*

*
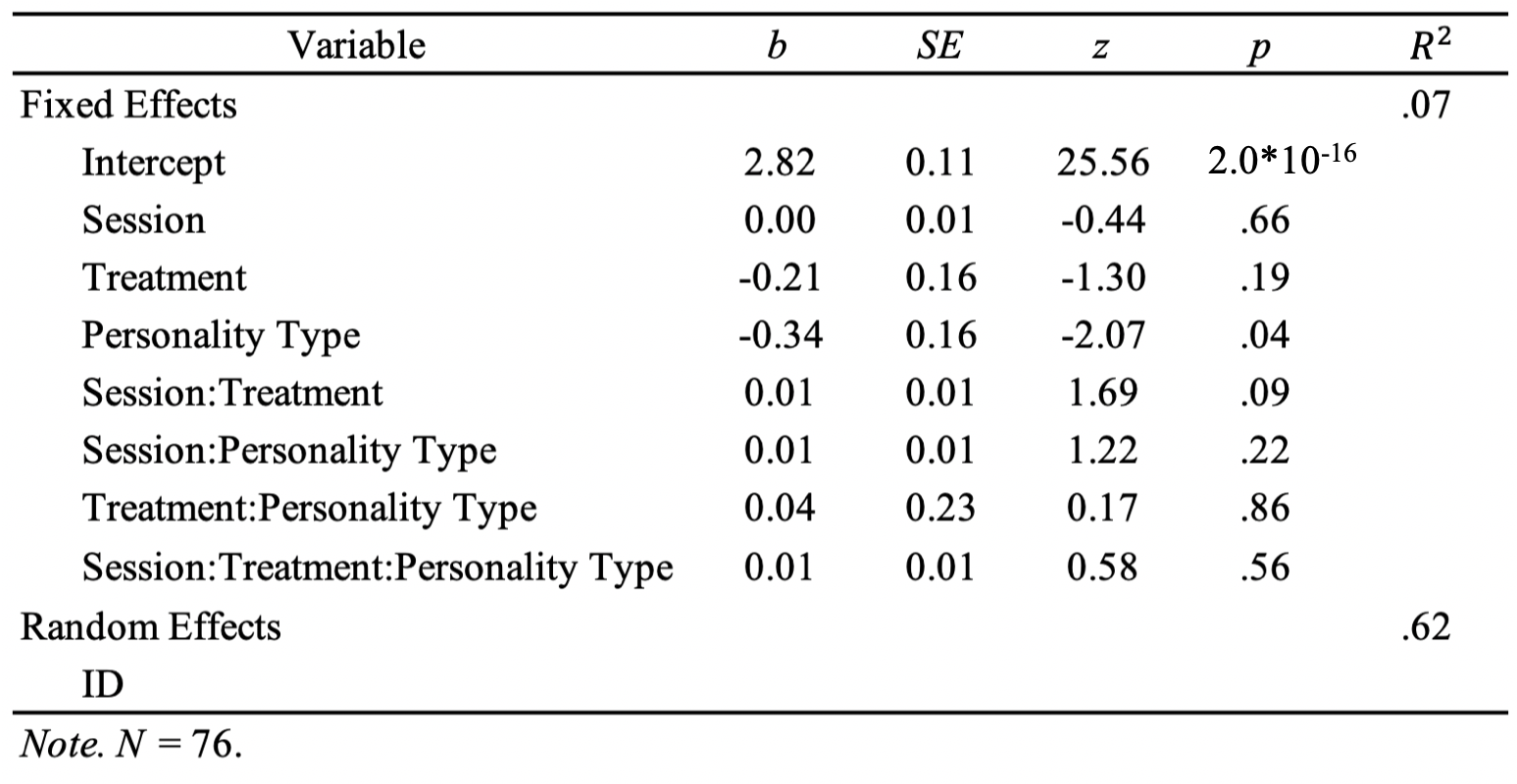
*

**Supplementary Table 6**

*Simple slopes of negative binomial mixed effect model of session, treatment, and personality type on the number of choices made in the 2-choice discrimination task*

**

**Supplementary Table 7**

*Linear mixed effect model of session and treatment, on the number of correct choices made in the 2-choice discrimination task* *pilot***
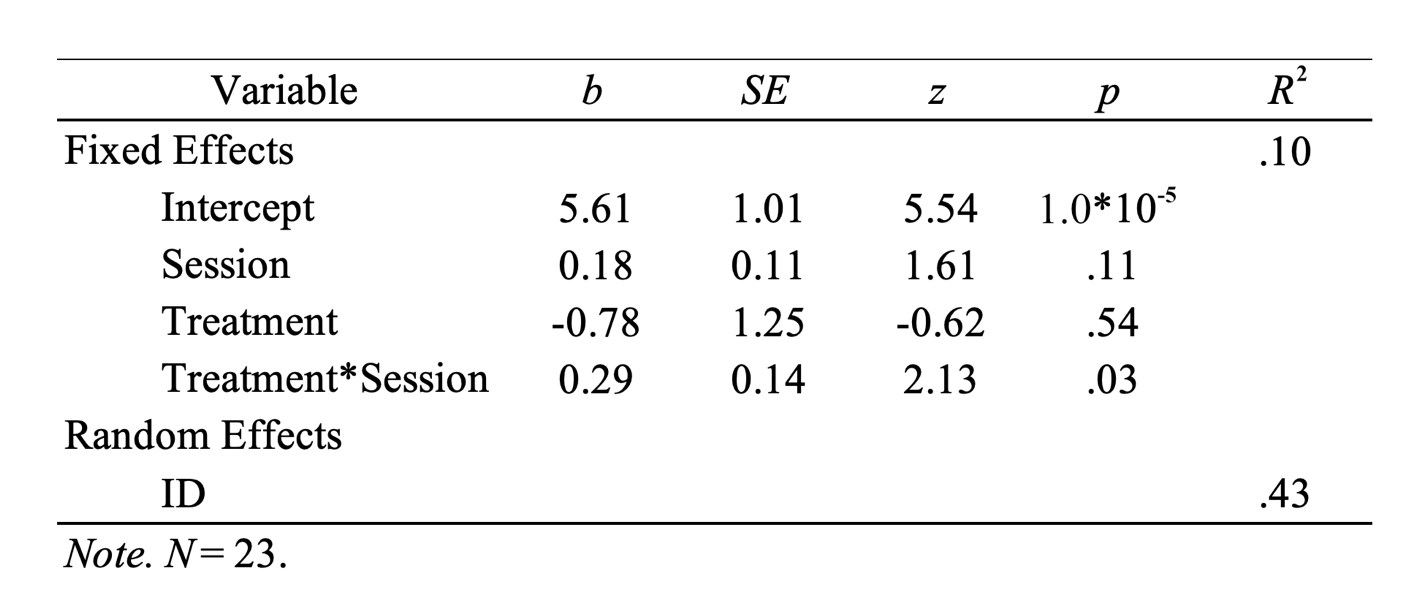
**

**Supplementary Table 8**

*All data from the OFT (freezing time) and motivation tasks (Time spent around food)*

| ID | Personality | Sex | Freezing Time | Time Spent Around Food |
| --- | --- | --- | --- | --- |
| BC1 | Bold | Female | 0.033367 | 45.049 |
| BC10 | Bold | Male | 0 | 94.866 |
| BC11 | Bold | Female | 0.033367 | 11.941 |
| BC12 | Bold | Male | 0 | 108.208 |
| BC13 | Bold | Male | 52.4189 | 84.609 |
| BC14 | Bold | Female | 0 | 123.609 |
| BC17 | Bold | Female | 128.495 | 91.95 |
| BC18 | Bold | Male | 0 | 74.957 |
| BC19 | Bold | Female | 113.446 | 128.55 |
| BC2 | Bold | Male | 0 | 37.062 |
| BC20 | Bold | Female | 22.022 | 127.954 |
| BC22 | Bold | Male | 0 | 88.304 |
| BC23 | Bold | Female | 50.7506 | 53.419 |
| BC24 | Bold | Male | 22.4224 | 53.997 |
| BC4 | Bold | Female | 0.033367 | 119.788 |
| BC5 | Bold | Female | 0 | 99.917 |
| BC6 | Bold | Male | 205.305 | 113.306 |
| BC7 | Bold | Female | 53.52 | 118.723 |
| BC8 | Bold | Female | 0 | 110.024 |
| BC9 | Bold | Female | 152.686 | 81.348 |
| BT1 | Bold | Male | 173.84 | 51.31 |
| BT10 | Bold | Male | 0.033367 | 80.29 |
| BT11 | Bold | Female | 97.8309 | 132.829 |
| BT12 | Bold | Male | 0.033367 | 125.271 |
| BT13 | Bold | Female | 0.233567 | 109.189 |
| BT14 | Bold | Male | 164.998 | 126.876 |
| BT15 | Bold | Male | 0.066733 | 126.851 |
| BT16 | Bold | Male | 61.0942 | 81.207 |
| BT17 | Bold | Female | 74.2741 | 120.059 |
| BT18 | Bold | Male | 79.2123 | 73.713 |
| BT2 | Bold | Female | 0.834164 | 72.911 |
| BT20 | Bold | Female | 25.9258 | 122.478 |
| BT3 | Bold | Male | 52.9194 | 106.374 |
| BT4 | Bold | Male | 0.066733 | 95.942 |
| BT5 | Bold | Female | 11.3446 | 60.953 |
| BT6 | Bold | Male | 0.033367 | 66.46 |
| BT7 | Bold | Male | 0.700699 | 96.73 |
| BT8 | Bold | Female | 57.4573 | 110.432 |
| BT9 | Bold | Female | 300.032 | 63.944 |
| SC1 | Shy | Male | 89.8563 | 76.09 |
| SC11 | Shy | Female | 295.294 | 85.713 |
| SC13 | Shy | Male | 300.032 | 122.75 |
| SC14 | Shy | Male | 0.033367 | 17.791 |
| SC15 | Shy | Female | 255.488 | 85.937 |
| SC16 | Shy | Female | 186.519 | 94.88 |
| SC17 | Shy | Female | 0.033367 | 119.279 |
| SC18 | Shy | Male | 246.012 | 91.49 |
| SC19 | Shy | Male | 1.001 | 135.548 |
| SC2 | Shy | Female | 35.4353 | 75.48 |
| SC20 | Shy | Male | 18.9856 | 105.158 |
| SC21 | Shy | Male | 18.7187 | 69.259 |
| SC22 | Shy | Female | 0 | 102.724 |
| SC23 | Shy | Female | 300.032 | 94.257 |
| SC25 | Shy | Male | 273.239 | 77.191 |
| SC26 | Shy | Female | 281.014 | 86.686 |
| SC27 | Shy | Male | 97.2636 | 29.795 |
| SC28 | Shy | Female | 244.444 | 79.212 |
| SC5 | Shy | Female | 0.033367 | 114.819 |
| SC6 | Shy | Male | 149.115 | 127.518 |
| SC7 | Shy | Male | 216.182 | 113.229 |
| SC8 | Shy | Male | 35.3352 | 40.529 |
| SC9 | Shy | Female | 300.032 | 105.071 |
| ST1 | Shy | Female | 0 | 115.378 |
| ST10 | Shy | Female | 40.2401 | 74.423 |
| ST11 | Shy | Male | 6.90689 | 89.056 |
| ST12 | Shy | Male | 28.8287 | 110.154 |
| ST13 | Shy | Female | 176.843 | 125.643 |
| ST14 | Shy | Male | 252.819 | 10 |
| ST16 | Shy | Female | 0 | 121.078 |
| ST17 | Shy | Male | 63.163 | 95.48 |
| ST18 | Shy | Male | 36.77 | 92.241 |
| ST2 | Shy | Male | 62.5624 | 124.834 |
| ST20 | Shy | Female | 134.901 | 41.216 |
| ST22 | Shy | Female | 82.1486 | 91.559 |
| ST23 | Shy | Female | 79.3791 | 93.958 |
| ST24 | Shy | Male | 33.7336 | 118.375 |
| ST3 | Shy | Female | 268.468 | 148.113 |
| ST4 | Shy | Female | 0.1001 | 89.966 |
| ST5 | Shy | Female | 294.66 | 81.584 |
| ST6 | Shy | Male | 44.3442 | 127.141 |
| ST7 | Shy | Male | 179.746 | 82.758 |
| ST8 | Shy | Female | 135.368 | 123.247 |
| ST9 | Shy | Female | 20.3203 | 102.015 |
| *N* = 83 |  |  |  |  |

**Supplementary Table 9**

*Raw data for time spent in the conditioned place preference task (sec)*

| ID | Sex | Treatment | Personality | Baseline | Probe 1 | Probe 2 | Probe 3 |
| --- | --- | --- | --- | --- | --- | --- | --- |
| ST1 | Female | Treatment | Shy | 118.356 | 167.859 | 156.79 | 170.373 |
| ST3 | Female | Treatment | Shy | 147.203 | 134.435 | 215.419 | 217.203 |
| SC1 | Male | Control | Shy | 33.365 | 74.455 | 205.325 | 76.576 |
| BT1 | Male | Treatment | Bold | 117.133 | 178.473 | 156.102 | 152.169 |
| BT2 | Female | Treatment | Bold | 177.2 | 193.754 | 197.027 | 162.666 |
| BT3 | Male | Treatment | Bold | 141.372 | 142.174 | 202.479 | 167.382 |
| BC1 | Female | Control | Bold | 140.055 | 84.717 | 115.531 | 132.806 |
| BC2 | Male | Control | Bold | 141.167 | 122.529 | 143.841 | 99.22 |
| ST4 | Female | Treatment | Shy | 114.463 | 165.459 | 264.738 | 205.165 |
| ST5 | Female | Treatment | Shy | 119.155 | 74.391 | 126.521 | 142.583 |
| ST6 | Male | Treatment | Shy | 121.51 | 133.055 | 126.08 | 174.181 |
| SC5 | Female | Control | Shy | 112.525 | 247.877 | 123.906 | 152.907 |
| SC6 | Male | Control | Shy | 140.549 | 134.546 | 142.488 | 113.93 |
| BT4 | Male | Treatment | Bold | 97.039 | 177.407 | 150.149 | 142.676 |
| BT5 | Female | Treatment | Bold | 109.957 | 108.608 | 115.759 | 131.713 |
| BT6 | Male | Treatment | Bold | 85.642 | 77.925 | 143.839 | 122.7 |
| BC4 | Female | Control | Bold | 130.243 | 150.034 | 160.655 | 95.255 |
| BC5 | Female | Control | Bold | 70.293 | 67.122 | 132.57 | 137.366 |
| BC6 | Male | Control | Bold | 68.851 | 45.391 | 83.708 | 106.859 |
| ST9 | Female | Treatment | Shy | 65.218 | 72.75 | 88.456 | 92.473 |
| SC7 | Male | Control | Shy | 139.45 | 88.103 | 64.659 | 138.895 |
| SC8 | Male | Control | Shy | 92.568 | 118.836 | 115.891 | 128.546 |
| BT7 | Male | Treatment | Bold | 54.856 | 102.651 | 117.082 | 146.469 |
| BT8 | Female | Treatment | Bold | 108.222 | 99.399 | 135.24 | 162.639 |
| BT9 | Female | Treatment | Bold | 202.98 | 235.885 | 190.293 | 165.422 |
| BC7 | Female | Control | Bold | 66.657 | 69.703 | 122.277 | 101.838 |
| BC8 | Female | Control | Bold | 166.412 | 125.91 | 137.729 | 141.336 |
| BC9 | Female | Control | Bold | 162.312 | 145.641 | 165.915 | 117.24 |
| ST10 | Female | Treatment | Shy | 108.703 | 45.626 | 142.671 | 114.609 |
| ST11 | Male | Treatment | Shy | 135.954 | 163.689 | 169.697 | 180.261 |
| ST12 | Male | Treatment | Shy | 96.505 | 127.223 | 154.823 | 172.177 |
| SC11 | Female | Control | Shy | 147.771 | 131 | 141.07 | 130.281 |
| BT10 | Male | Treatment | Bold | 135.743 | 125.695 | 119.338 | 142.121 |
| BT11 | Female | Treatment | Bold | 124.131 | 166.217 | 159.934 | 144.81 |
| BT12 | Male | Treatment | Bold | 60.948 | 205.826 | 190.957 | 186.584 |
| BC10 | Male | Control | Bold | 135.704 | 145.208 | 163.974 | 133.696 |
| BC11 | Female | Control | Bold | 114.083 | 171.706 | 160.179 | 114.034 |
| BC12 | Male | Control | Bold | 112.347 | 150.701 | 117.825 | 108.041 |
| ST14 | Male | Treatment | Shy | 27.834 | 25.33 | 77.177 | 124.83 |
| ST13 | Female | Treatment | Shy | 131.843 | 128.339 | 143.367 | 161.243 |
| SC13 | Male | Control | Shy | 112.609 | 188.834 | 151.743 | 98.931 |
| SC15 | Female | Control | Shy | 212.314 | 185.564 | 193.945 | 126.232 |
| BT13 | Female | Treatment | Bold | 107.908 | 156.702 | 126.032 | 192.285 |
| BT14 | Male | Treatment | Bold | 136.803 | 162.996 | 191.926 | 211.289 |
| BT15 | Male | Treatment | Bold | 129.507 | 156.97 | 228.362 | 212.238 |
| BC13 | Male | Control | Bold | 149.51 | 230.757 | 176.963 | 171.335 |
| BC14 | Female | Control | Bold | 134.042 | 82.496 | 126.995 | 132.19 |
| ST16 | Female | Treatment | Shy | 99.808 | 167.519 | 167.519 | 173.633 |
| ST17 | Male | Treatment | Shy | 120.716 | 168.293 | 43.912 | 82.453 |
| ST18 | Male | Treatment | Shy | 120.644 | 171.291 | 179.023 | 113.762 |
| SC16 | Female | Control | Shy | 127.238 | 96.376 | 259.843 | 43.089 |
| SC17 | Female | Control | Shy | 134.771 | 136.753 | 91.129 | 128.806 |
| SC18 | Male | Control | Shy | 97.324 | 79.507 | 40.164 | 133.753 |
| BT16 | Male | Treatment | Bold | 114.296 | 157.99 | 232.114 | 186.234 |
| BT17 | Female | Treatment | Bold | 112.544 | 173.333 | 204.514 | 199.99 |
| BT18 | Male | Treatment | Bold | 55.743 | 140.809 | 148.286 | 126.616 |
| BC17 | Female | Control | Bold | 122.383 | 91.207 | 144.717 | 122.392 |
| BC18 | Male | Control | Bold | 244.269 | 159.195 | 151.71 | 173.382 |
| ST19 | Female | Treatment | Shy | 139.607 | 224.92 | 224.92 | 224.92 |
| ST20 | Female | Treatment | Shy | 125.143 | 109.68 | 109.68 | 109.68 |
| SC19 | Male | Control | Shy | 0 | 51.891 | 51.891 | 51.891 |
| SC20 | Male | Control | Shy | 35.305 | 21.867 | 21.867 | 21.867 |
| SC21 | Male | Control | Shy | 101.512 | 111.113 | 111.113 | 111.113 |
| SC22 | Female | Control | Shy | 125.936 | 56.558 | 56.558 | 56.558 |
| BC23 | Female | Control | Bold | 120.736 | 116.22 | 116.22 | 116.22 |
| BC24 | Male | Control | Bold | 127.405 | 75.513 | 75.513 | 75.513 |
| BC19 | Female | Control | Bold | 112.307 | 160.022 | 160.022 | 160.022 |
| BC20 | Female | Control | Bold | 105.107 | 170.818 | 170.818 | 170.818 |
| BT20 | Female | Treatment | Bold | 145.56 | 171.943 | 171.943 | 171.943 |
| BC22 | Male | Control | Bold | 129.533 | 156.04 | 156.04 | 156.04 |
| SC23 | Female | Control | Shy | 51.278 | 22.902 | 22.902 | 22.902 |
| SC25 | Male | Control | Shy | 138.839 | 169.031 | 169.031 | 169.031 |
| SC26 | Female | Control | Shy | 137.818 | 165.949 | 165.949 | 165.949 |
| SC27 | Male | Control | Shy | 144.089 | 126.778 | 126.778 | 126.778 |
| SC28 | Female | Control | Shy | 143.824 | 35.418 | 35.418 | 35.418 |
| ST22 | Female | Treatment | Shy | 110.911 | 129.745 | 129.745 | 129.745 |
| ST23 | Female | Treatment | Shy | 145.934 | 140.214 | 140.214 | 140.214 |
| ST24 | Male | Treatment | Shy | 144.729 | 190.334 | 190.334 | 190.334 |
| *N* = 78 |  |  |  |  |  |  |  |

**Supplementary Table 10**

*Number of correct, incorrect, and total choices made in the 2-choice discrimination task*

| ID | Sex | Treatment | Personality | Session | Correct | Incorrect | Total Choices |
| --- | --- | --- | --- | --- | --- | --- | --- |
| ST1 | Female | Treatment | Shy | 1 | 1 | 0 | 1 |
| ST1 | Female | Treatment | Shy | 2 | 1 | 1 | 2 |
| ST1 | Female | Treatment | Shy | 3 | 2 | 2 | 4 |
| ST1 | Female | Treatment | Shy | 4 | 2 | 1 | 3 |
| ST1 | Female | Treatment | Shy | 5 | 3 | 0 | 3 |
| ST1 | Female | Treatment | Shy | 6 | 2 | 2 | 4 |
| ST1 | Female | Treatment | Shy | 8 | 3 | 0 | 3 |
| ST1 | Female | Treatment | Shy | 9 | 2 | 2 | 4 |
| ST1 | Female | Treatment | Shy | 10 | 2 | 0 | 2 |
| ST2 | Male | Treatment | Shy | 1 | 2 | 0 | 2 |
| ST2 | Male | Treatment | Shy | 2 | 4 | 0 | 4 |
| ST2 | Male | Treatment | Shy | 3 | 4 | 1 | 5 |
| ST2 | Male | Treatment | Shy | 4 | 7 | 0 | 7 |
| ST2 | Male | Treatment | Shy | 5 | 7 | 6 | 13 |
| ST2 | Male | Treatment | Shy | 6 | 8 | 4 | 12 |
| ST2 | Male | Treatment | Shy | 7 | 3 | 12 | 15 |
| ST2 | Male | Treatment | Shy | 8 | 8 | 12 | 20 |
| ST2 | Male | Treatment | Shy | 9 | 8 | 2 | 10 |
| ST2 | Male | Treatment | Shy | 10 | 7 | 8 | 15 |
| ST3 | Female | Treatment | Shy | 1 | 7 | 13 | 20 |
| ST3 | Female | Treatment | Shy | 2 | 11 | 9 | 20 |
| ST3 | Female | Treatment | Shy | 3 | 9 | 10 | 19 |
| ST3 | Female | Treatment | Shy | 4 | 16 | 4 | 20 |
| ST3 | Female | Treatment | Shy | 5 | 13 | 7 | 20 |
| ST3 | Female | Treatment | Shy | 6 | 6 | 13 | 19 |
| ST3 | Female | Treatment | Shy | 7 | 8 | 12 | 20 |
| ST3 | Female | Treatment | Shy | 8 | 7 | 12 | 19 |
| ST3 | Female | Treatment | Shy | 9 | 11 | 9 | 20 |
| ST3 | Female | Treatment | Shy | 10 | 11 | 7 | 18 |
| SC1 | Male | Control | Shy | 1 | 0 | 1 | 1 |
| SC1 | Male | Control | Shy | 2 | 1 | 0 | 1 |
| SC1 | Male | Control | Shy | 3 | 11 | 5 | 16 |
| SC1 | Male | Control | Shy | 4 | 15 | 5 | 20 |
| SC1 | Male | Control | Shy | 5 | 12 | 7 | 19 |
| SC1 | Male | Control | Shy | 6 | 14 | 5 | 19 |
| SC1 | Male | Control | Shy | 7 | 14 | 1 | 15 |
| SC1 | Male | Control | Shy | 8 | 8 | 7 | 15 |
| SC1 | Male | Control | Shy | 9 | 7 | 8 | 15 |
| SC1 | Male | Control | Shy | 10 | 12 | 7 | 19 |
| SC2 | Female | Control | Shy | 1 | 14 | 5 | 19 |
| SC2 | Female | Control | Shy | 2 | 6 | 14 | 20 |
| SC2 | Female | Control | Shy | 3 | 10 | 10 | 20 |
| SC2 | Female | Control | Shy | 4 | 9 | 10 | 19 |
| SC2 | Female | Control | Shy | 5 | 7 | 10 | 17 |
| SC2 | Female | Control | Shy | 6 | 12 | 6 | 18 |
| SC2 | Female | Control | Shy | 7 | 10 | 5 | 15 |
| SC2 | Female | Control | Shy | 8 | 11 | 7 | 18 |
| SC2 | Female | Control | Shy | 9 | 12 | 7 | 19 |
| SC2 | Female | Control | Bold | 10 | 6 | 11 | 17 |
| BT1 | Male | Treatment | Bold | 1 | 13 | 5 | 18 |
| BT1 | Male | Treatment | Bold | 2 | 10 | 10 | 20 |
| BT1 | Male | Treatment | Bold | 3 | 12 | 8 | 20 |
| BT1 | Male | Treatment | Bold | 4 | 9 | 10 | 19 |
| BT1 | Male | Treatment | Bold | 5 | 6 | 10 | 16 |
| BT1 | Male | Treatment | Bold | 6 | 8 | 10 | 18 |
| BT1 | Male | Treatment | Bold | 7 | 13 | 5 | 18 |
| BT1 | Male | Treatment | Bold | 8 | 5 | 5 | 10 |
| BT1 | Male | Treatment | Bold | 9 | 8 | 8 | 16 |
| BT1 | Male | Treatment | Bold | 10 | 1 | 5 | 6 |
| BT2 | Female | Treatment | Bold | 1 | 2 | 0 | 2 |
| BT2 | Female | Treatment | Bold | 2 | 15 | 1 | 16 |
| BT2 | Female | Treatment | Bold | 3 | 11 | 8 | 19 |
| BT2 | Female | Treatment | Bold | 4 | 13 | 7 | 20 |
| BT2 | Female | Treatment | Bold | 5 | 13 | 7 | 20 |
| BT2 | Female | Treatment | Bold | 6 | 11 | 8 | 19 |
| BT2 | Female | Treatment | Bold | 7 | 11 | 8 | 19 |
| BT2 | Female | Treatment | Bold | 8 | 12 | 8 | 20 |
| BT2 | Female | Treatment | Bold | 9 | 10 | 10 | 20 |
| BT2 | Female | Treatment | Bold | 10 | 11 | 9 | 20 |
| BT3 | Male | Treatment | Bold | 1 | 5 | 10 | 15 |
| BT3 | Male | Treatment | Bold | 2 | 4 | 6 | 10 |
| BT3 | Male | Treatment | Bold | 3 | 3 | 5 | 8 |
| BT3 | Male | Treatment | Bold | 4 | 3 | 7 | 10 |
| BT3 | Male | Treatment | Bold | 5 | 3 | 9 | 12 |
| BT3 | Male | Treatment | Bold | 6 | 5 | 6 | 11 |
| BT3 | Male | Treatment | Bold | 7 | 4 | 3 | 7 |
| BT3 | Male | Treatment | Bold | 8 | 10 | 9 | 19 |
| BT3 | Male | Treatment | Bold | 9 | 10 | 10 | 20 |
| BT3 | Male | Treatment | Bold | 10 | 12 | 5 | 17 |
| BC1 | Female | Control | Bold | 1 | 7 | 13 | 20 |
| BC1 | Female | Control | Bold | 2 | 9 | 10 | 19 |
| BC1 | Female | Control | Bold | 3 | 12 | 7 | 19 |
| BC1 | Female | Control | Bold | 4 | 8 | 10 | 18 |
| BC1 | Female | Control | Bold | 5 | 11 | 6 | 17 |
| BC1 | Female | Control | Bold | 6 | 11 | 4 | 15 |
| BC1 | Female | Control | Bold | 7 | 7 | 7 | 14 |
| BC1 | Female | Control | Bold | 8 | 7 | 8 | 15 |
| BC1 | Female | Control | Bold | 9 | 11 | 4 | 15 |
| BC1 | Female | Control | Bold | 10 | 13 | 7 | 20 |
| BC2 | Male | Control | Bold | 1 | 10 | 10 | 20 |
| BC2 | Male | Control | Bold | 2 | 11 | 6 | 17 |
| BC2 | Male | Control | Bold | 3 | 11 | 8 | 19 |
| BC2 | Male | Control | Bold | 4 | 16 | 3 | 19 |
| BC2 | Male | Control | Bold | 5 | 9 | 11 | 20 |
| BC2 | Male | Control | Bold | 6 | 12 | 8 | 20 |
| BC2 | Male | Control | Bold | 7 | 13 | 7 | 20 |
| BC2 | Male | Control | Bold | 8 | 10 | 10 | 20 |
| BC2 | Male | Control | Bold | 9 | 12 | 8 | 20 |
| BC2 | Male | Control | Shy | 10 | 9 | 10 | 19 |
| ST4 | Female | Treatment | Shy | 1 | 9 | 10 | 19 |
| ST4 | Female | Treatment | Shy | 2 | 8 | 12 | 20 |
| ST4 | Female | Treatment | Shy | 3 | 10 | 10 | 20 |
| ST4 | Female | Treatment | Shy | 4 | 7 | 13 | 20 |
| ST4 | Female | Treatment | Shy | 5 | 12 | 8 | 20 |
| ST4 | Female | Treatment | Shy | 6 | 6 | 14 | 20 |
| ST4 | Female | Treatment | Shy | 7 | 10 | 10 | 20 |
| ST4 | Female | Treatment | Shy | 8 | 8 | 12 | 20 |
| ST4 | Female | Treatment | Shy | 9 | 12 | 6 | 18 |
| ST4 | Female | Treatment | Shy | 10 | 8 | 12 | 20 |
| ST5 | Female | Treatment | Shy | 1 | 6 | 14 | 20 |
| ST5 | Female | Treatment | Shy | 2 | 11 | 9 | 20 |
| ST5 | Female | Treatment | Shy | 3 | 10 | 10 | 20 |
| ST5 | Female | Treatment | Shy | 4 | 5 | 15 | 20 |
| ST5 | Female | Treatment | Shy | 5 | 12 | 8 | 20 |
| ST5 | Female | Treatment | Shy | 6 | 12 | 8 | 20 |
| ST5 | Female | Treatment | Shy | 7 | 9 | 11 | 20 |
| ST5 | Female | Treatment | Shy | 8 | 5 | 15 | 20 |
| ST5 | Female | Treatment | Shy | 9 | 9 | 11 | 20 |
| ST5 | Female | Treatment | Shy | 10 | 8 | 12 | 20 |
| ST6 | Male | Treatment | Shy | 1 | 5 | 2 | 7 |
| ST6 | Male | Treatment | Shy | 2 | 4 | 3 | 7 |
| ST6 | Male | Treatment | Shy | 3 | 12 | 2 | 14 |
| ST6 | Male | Treatment | Shy | 4 | 8 | 5 | 13 |
| ST6 | Male | Treatment | Shy | 5 | 9 | 7 | 16 |
| ST6 | Male | Treatment | Shy | 6 | 9 | 5 | 14 |
| ST6 | Male | Treatment | Shy | 7 | 5 | 8 | 13 |
| ST6 | Male | Treatment | Shy | 8 | 11 | 7 | 18 |
| ST6 | Male | Treatment | Shy | 9 | 11 | 6 | 17 |
| ST6 | Male | Treatment | Shy | 10 | 8 | 9 | 17 |
| SC6 | Male | Control | Shy | 1 | 8 | 11 | 19 |
| SC6 | Male | Control | Shy | 2 | 7 | 11 | 18 |
| SC6 | Male | Control | Shy | 3 | 8 | 12 | 20 |
| SC6 | Male | Control | Shy | 4 | 5 | 14 | 19 |
| SC6 | Male | Control | Shy | 5 | 10 | 10 | 20 |
| SC6 | Male | Control | Shy | 6 | 5 | 11 | 16 |
| SC6 | Male | Control | Shy | 7 | 13 | 4 | 17 |
| SC6 | Male | Control | Shy | 8 | 13 | 5 | 18 |
| SC6 | Male | Control | Bold | 9 | 11 | 9 | 20 |
| BT4 | Male | Treatment | Bold | 1 | 8 | 2 | 10 |
| BT4 | Male | Treatment | Bold | 2 | 11 | 6 | 17 |
| BT4 | Male | Treatment | Bold | 3 | 11 | 8 | 19 |
| BT4 | Male | Treatment | Bold | 4 | 6 | 10 | 16 |
| BT4 | Male | Treatment | Bold | 5 | 13 | 7 | 20 |
| BT4 | Male | Treatment | Bold | 6 | 11 | 8 | 19 |
| BT4 | Male | Treatment | Bold | 7 | 7 | 11 | 18 |
| BT4 | Male | Treatment | Bold | 8 | 5 | 11 | 16 |
| BT4 | Male | Treatment | Bold | 9 | 4 | 16 | 20 |
| BT4 | Male | Treatment | Bold | 10 | 7 | 11 | 18 |
| BT5 | Female | Treatment | Bold | 1 | 10 | 10 | 20 |
| BT5 | Female | Treatment | Bold | 2 | 9 | 9 | 18 |
| BT5 | Female | Treatment | Bold | 3 | 8 | 12 | 20 |
| BT5 | Female | Treatment | Bold | 4 | 12 | 8 | 20 |
| BT5 | Female | Treatment | Bold | 5 | 4 | 16 | 20 |
| BT5 | Female | Treatment | Bold | 6 | 7 | 13 | 20 |
| BT5 | Female | Treatment | Bold | 7 | 5 | 15 | 20 |
| BT5 | Female | Treatment | Bold | 8 | 10 | 10 | 20 |
| BT5 | Female | Treatment | Bold | 9 | 7 | 12 | 19 |
| BT5 | Female | Treatment | Bold | 10 | 6 | 14 | 20 |
| BT6 | Male | Treatment | Bold | 1 | 8 | 9 | 17 |
| BT6 | Male | Treatment | Bold | 2 | 11 | 5 | 16 |
| BT6 | Male | Treatment | Bold | 3 | 10 | 5 | 15 |
| BT6 | Male | Treatment | Bold | 4 | 7 | 7 | 14 |
| BT6 | Male | Treatment | Bold | 5 | 7 | 11 | 18 |
| BT6 | Male | Treatment | Bold | 6 | 11 | 9 | 20 |
| BT6 | Male | Treatment | Bold | 7 | 10 | 10 | 20 |
| BT6 | Male | Treatment | Bold | 8 | 11 | 6 | 17 |
| BT6 | Male | Treatment | Bold | 9 | 12 | 7 | 19 |
| BT6 | Male | Treatment | Bold | 10 | 8 | 9 | 17 |
| BC4 | Female | Control | Bold | 1 | 9 | 9 | 18 |
| BC4 | Female | Control | Bold | 2 | 8 | 11 | 19 |
| BC4 | Female | Control | Bold | 3 | 11 | 8 | 19 |
| BC4 | Female | Control | Bold | 4 | 8 | 11 | 19 |
| BC4 | Female | Control | Bold | 5 | 14 | 6 | 20 |
| BC4 | Female | Control | Bold | 6 | 6 | 12 | 18 |
| BC4 | Female | Control | Bold | 7 | 10 | 8 | 18 |
| BC4 | Female | Control | Bold | 8 | 9 | 10 | 19 |
| BC4 | Female | Control | Bold | 9 | 7 | 11 | 18 |
| BC4 | Female | Control | Bold | 10 | 6 | 11 | 17 |
| BC5 | Female | Control | Bold | 1 | 9 | 11 | 20 |
| BC5 | Female | Control | Bold | 2 | 9 | 10 | 19 |
| BC5 | Female | Control | Bold | 3 | 11 | 5 | 16 |
| BC5 | Female | Control | Bold | 4 | 5 | 7 | 12 |
| BC5 | Female | Control | Bold | 5 | 12 | 5 | 17 |
| BC5 | Female | Control | Bold | 6 | 9 | 5 | 14 |
| BC5 | Female | Control | Bold | 7 | 7 | 13 | 20 |
| BC5 | Female | Control | Bold | 8 | 10 | 7 | 17 |
| BC5 | Female | Control | Bold | 9 | 13 | 6 | 19 |
| BC5 | Female | Control | Bold | 10 | 5 | 10 | 15 |
| BC6 | Male | Control | Bold | 1 | 8 | 10 | 18 |
| BC6 | Male | Control | Bold | 2 | 12 | 7 | 19 |
| BC6 | Male | Control | Bold | 3 | 4 | 8 | 12 |
| BC6 | Male | Control | Bold | 4 | 7 | 7 | 14 |
| BC6 | Male | Control | Bold | 5 | 6 | 13 | 19 |
| BC6 | Male | Control | Bold | 6 | 6 | 7 | 13 |
| BC6 | Male | Control | Bold | 7 | 12 | 6 | 18 |
| BC6 | Male | Control | Bold | 8 | 9 | 9 | 18 |
| BC6 | Male | Control | Bold | 9 | 7 | 12 | 19 |
| BC6 | Male | Control | Shy | 10 | 14 | 4 | 18 |
| ST7 | Male | Treatment | Shy | 1 | 2 | 4 | 6 |
| ST7 | Male | Treatment | Shy | 2 | 14 | 3 | 17 |
| ST7 | Male | Treatment | Shy | 3 | 6 | 11 | 17 |
| ST7 | Male | Treatment | Shy | 4 | 13 | 6 | 19 |
| ST7 | Male | Treatment | Shy | 5 | 12 | 5 | 17 |
| ST7 | Male | Treatment | Shy | 6 | 12 | 8 | 20 |
| ST7 | Male | Treatment | Shy | 7 | 6 | 13 | 19 |
| ST7 | Male | Treatment | Shy | 8 | 6 | 14 | 20 |
| ST7 | Male | Treatment | Shy | 9 | 9 | 4 | 13 |
| ST7 | Male | Treatment | Shy | 10 | 11 | 8 | 19 |
| ST8 | Female | Treatment | Shy | 1 | 2 | 3 | 5 |
| ST8 | Female | Treatment | Shy | 2 | 2 | 6 | 8 |
| ST8 | Female | Treatment | Shy | 3 | 7 | 5 | 12 |
| ST8 | Female | Treatment | Shy | 4 | 6 | 10 | 16 |
| ST8 | Female | Treatment | Shy | 5 | 8 | 9 | 17 |
| ST8 | Female | Treatment | Shy | 6 | 6 | 7 | 13 |
| ST8 | Female | Treatment | Shy | 7 | 5 | 5 | 10 |
| ST8 | Female | Treatment | Shy | 8 | 8 | 10 | 18 |
| ST8 | Female | Treatment | Shy | 9 | 8 | 6 | 14 |
| ST8 | Female | Treatment | Shy | 10 | 0 | 1 | 1 |
| ST9 | Female | Treatment | Shy | 1 | 4 | 1 | 5 |
| ST9 | Female | Treatment | Shy | 2 | 0 | 6 | 6 |
| ST9 | Female | Treatment | Shy | 3 | 0 | 0 | 0 |
| ST9 | Female | Treatment | Shy | 4 | 0 | 3 | 3 |
| ST9 | Female | Treatment | Shy | 5 | 0 | 0 | 0 |
| ST9 | Female | Treatment | Shy | 6 | 1 | 9 | 10 |
| ST9 | Female | Treatment | Shy | 7 | 0 | 1 | 1 |
| ST9 | Female | Treatment | Shy | 8 | 8 | 1 | 9 |
| ST9 | Female | Treatment | Shy | 9 | 0 | 2 | 2 |
| ST9 | Female | Treatment | Shy | 10 | 0 | 1 | 1 |
| SC7 | Male | Control | Shy | 1 | 8 | 12 | 20 |
| SC7 | Male | Control | Shy | 2 | 11 | 9 | 20 |
| SC7 | Male | Control | Shy | 3 | 4 | 8 | 12 |
| SC7 | Male | Control | Shy | 4 | 9 | 11 | 20 |
| SC7 | Male | Control | Shy | 5 | 10 | 10 | 20 |
| SC7 | Male | Control | Shy | 6 | 8 | 12 | 20 |
| SC7 | Male | Control | Shy | 7 | 6 | 13 | 19 |
| SC7 | Male | Control | Shy | 8 | 10 | 10 | 20 |
| SC7 | Male | Control | Shy | 9 | 12 | 8 | 20 |
| SC7 | Male | Control | Shy | 10 | 11 | 9 | 20 |
| SC8 | Male | Control | Shy | 1 | 14 | 5 | 19 |
| SC8 | Male | Control | Shy | 2 | 11 | 4 | 15 |
| SC8 | Male | Control | Shy | 3 | 13 | 7 | 20 |
| SC8 | Male | Control | Shy | 4 | 10 | 10 | 20 |
| SC8 | Male | Control | Shy | 5 | 8 | 10 | 18 |
| SC8 | Male | Control | Shy | 6 | 9 | 9 | 18 |
| SC8 | Male | Control | Shy | 7 | 9 | 8 | 17 |
| SC8 | Male | Control | Shy | 8 | 8 | 8 | 16 |
| SC8 | Male | Control | Shy | 9 | 13 | 2 | 15 |
| SC8 | Male | Control | Bold | 10 | 9 | 5 | 14 |
| BT7 | Male | Treatment | Bold | 1 | 12 | 6 | 18 |
| BT7 | Male | Treatment | Bold | 2 | 12 | 6 | 18 |
| BT7 | Male | Treatment | Bold | 3 | 9 | 8 | 17 |
| BT7 | Male | Treatment | Bold | 4 | 8 | 8 | 16 |
| BT7 | Male | Treatment | Bold | 5 | 6 | 6 | 12 |
| BT7 | Male | Treatment | Bold | 6 | 8 | 3 | 11 |
| BT7 | Male | Treatment | Bold | 7 | 5 | 7 | 12 |
| BT7 | Male | Treatment | Bold | 8 | 8 | 6 | 14 |
| BT7 | Male | Treatment | Bold | 9 | 8 | 6 | 14 |
| BT7 | Male | Treatment | Bold | 10 | 11 | 8 | 19 |
| BT8 | Female | Treatment | Bold | 1 | 5 | 1 | 6 |
| BT8 | Female | Treatment | Bold | 2 | 4 | 2 | 6 |
| BT8 | Female | Treatment | Bold | 3 | 3 | 1 | 4 |
| BT8 | Female | Treatment | Bold | 4 | 7 | 0 | 7 |
| BT8 | Female | Treatment | Bold | 5 | 5 | 0 | 5 |
| BT8 | Female | Treatment | Bold | 6 | 14 | 2 | 16 |
| BT8 | Female | Treatment | Bold | 7 | 9 | 2 | 11 |
| BT8 | Female | Treatment | Bold | 8 | 13 | 3 | 16 |
| BT8 | Female | Treatment | Bold | 9 | 14 | 4 | 18 |
| BT8 | Female | Treatment | Bold | 10 | 9 | 6 | 15 |
| BT9 | Female | Treatment | Bold | 1 | 1 | 3 | 4 |
| BT9 | Female | Treatment | Bold | 2 | 0 | 2 | 2 |
| BT9 | Female | Treatment | Bold | 3 | 1 | 4 | 5 |
| BT9 | Female | Treatment | Bold | 4 | 0 | 0 | 0 |
| BT9 | Female | Treatment | Bold | 5 | 0 | 1 | 1 |
| BT9 | Female | Treatment | Bold | 6 | 0 | 0 | 0 |
| BT9 | Female | Treatment | Bold | 7 | 0 | 3 | 3 |
| BT9 | Female | Treatment | Bold | 8 | 1 | 2 | 3 |
| BT9 | Female | Treatment | Bold | 9 | 1 | 2 | 3 |
| BT9 | Female | Treatment | Bold | 10 | 1 | 2 | 3 |
| BC7 | Female | Control | Bold | 1 | 11 | 8 | 19 |
| BC7 | Female | Control | Bold | 2 | 12 | 6 | 18 |
| BC7 | Female | Control | Bold | 3 | 9 | 6 | 15 |
| BC7 | Female | Control | Bold | 4 | 10 | 9 | 19 |
| BC7 | Female | Control | Bold | 5 | 12 | 7 | 19 |
| BC7 | Female | Control | Bold | 6 | 4 | 6 | 10 |
| BC7 | Female | Control | Bold | 7 | 6 | 4 | 10 |
| BC7 | Female | Control | Bold | 8 | 6 | 8 | 14 |
| BC7 | Female | Control | Bold | 9 | 8 | 6 | 14 |
| BC7 | Female | Control | Bold | 10 | 5 | 5 | 10 |
| BC8 | Female | Control | Bold | 1 | 8 | 7 | 15 |
| BC8 | Female | Control | Bold | 2 | 11 | 7 | 18 |
| BC8 | Female | Control | Bold | 3 | 4 | 4 | 8 |
| BC8 | Female | Control | Bold | 4 | 5 | 7 | 12 |
| BC8 | Female | Control | Bold | 5 | 3 | 3 | 6 |
| BC8 | Female | Control | Bold | 6 | 9 | 4 | 13 |
| BC8 | Female | Control | Bold | 7 | 8 | 6 | 14 |
| BC8 | Female | Control | Bold | 8 | 7 | 2 | 9 |
| BC8 | Female | Control | Bold | 9 | 10 | 7 | 17 |
| BC8 | Female | Control | Bold | 10 | 5 | 5 | 10 |
| BC9 | Female | Control | Bold | 1 | 13 | 6 | 19 |
| BC9 | Female | Control | Bold | 2 | 8 | 12 | 20 |
| BC9 | Female | Control | Bold | 3 | 5 | 13 | 18 |
| BC9 | Female | Control | Bold | 4 | 10 | 9 | 19 |
| BC9 | Female | Control | Bold | 5 | 5 | 13 | 18 |
| BC9 | Female | Control | Bold | 6 | 8 | 11 | 19 |
| BC9 | Female | Control | Bold | 7 | 8 | 9 | 17 |
| BC9 | Female | Control | Bold | 8 | 11 | 9 | 20 |
| BC9 | Female | Control | Bold | 9 | 9 | 9 | 18 |
| BC9 | Female | Control | Shy | 10 | 6 | 12 | 18 |
| ST10 | Female | Treatment | Shy | 1 | 9 | 10 | 19 |
| ST10 | Female | Treatment | Shy | 2 | 8 | 10 | 18 |
| ST10 | Female | Treatment | Shy | 3 | 11 | 9 | 20 |
| ST10 | Female | Treatment | Shy | 4 | 8 | 9 | 17 |
| ST10 | Female | Treatment | Shy | 5 | 14 | 6 | 20 |
| ST10 | Female | Treatment | Shy | 6 | 11 | 9 | 20 |
| ST10 | Female | Treatment | Shy | 7 | 12 | 5 | 17 |
| ST10 | Female | Treatment | Shy | 8 | 12 | 7 | 19 |
| ST10 | Female | Treatment | Shy | 9 | 11 | 6 | 17 |
| ST10 | Female | Treatment | Shy | 10 | 12 | 7 | 19 |
| ST11 | Male | Treatment | Shy | 1 | 8 | 11 | 19 |
| ST11 | Male | Treatment | Shy | 2 | 6 | 13 | 19 |
| ST11 | Male | Treatment | Shy | 3 | 8 | 11 | 19 |
| ST11 | Male | Treatment | Shy | 4 | 6 | 12 | 18 |
| ST11 | Male | Treatment | Shy | 5 | 9 | 9 | 18 |
| ST11 | Male | Treatment | Shy | 6 | 5 | 13 | 18 |
| ST11 | Male | Treatment | Shy | 7 | 8 | 11 | 19 |
| ST11 | Male | Treatment | Shy | 8 | 4 | 15 | 19 |
| ST11 | Male | Treatment | Shy | 9 | 11 | 6 | 17 |
| ST11 | Male | Treatment | Bold | 10 | 5 | 10 | 15 |
| BT10 | Male | Treatment | Bold | 1 | 6 | 12 | 18 |
| BT10 | Male | Treatment | Bold | 2 | 13 | 7 | 20 |
| BT10 | Male | Treatment | Bold | 3 | 12 | 8 | 20 |
| BT10 | Male | Treatment | Bold | 4 | 10 | 8 | 18 |
| BT10 | Male | Treatment | Bold | 5 | 8 | 12 | 20 |
| BT10 | Male | Treatment | Bold | 6 | 8 | 11 | 19 |
| BT10 | Male | Treatment | Bold | 7 | 8 | 9 | 17 |
| BT10 | Male | Treatment | Bold | 8 | 6 | 10 | 16 |
| BT10 | Male | Treatment | Bold | 9 | 11 | 9 | 20 |
| BT10 | Male | Treatment | Bold | 10 | 7 | 7 | 14 |
| BT11 | Female | Treatment | Bold | 1 | 11 | 9 | 20 |
| BT11 | Female | Treatment | Bold | 2 | 9 | 9 | 18 |
| BT11 | Female | Treatment | Bold | 3 | 10 | 9 | 19 |
| BT11 | Female | Treatment | Bold | 4 | 11 | 9 | 20 |
| BT11 | Female | Treatment | Bold | 5 | 10 | 8 | 18 |
| BT11 | Female | Treatment | Bold | 6 | 6 | 11 | 17 |
| BT11 | Female | Treatment | Bold | 7 | 12 | 7 | 19 |
| BT11 | Female | Treatment | Bold | 8 | 13 | 5 | 18 |
| BT11 | Female | Treatment | Bold | 9 | 6 | 14 | 20 |
| BT11 | Female | Treatment | Bold | 10 | 13 | 6 | 19 |
| BT12 | Male | Treatment | Bold | 1 | 9 | 10 | 19 |
| BT12 | Male | Treatment | Bold | 2 | 7 | 8 | 15 |
| BT12 | Male | Treatment | Bold | 3 | 6 | 9 | 15 |
| BT12 | Male | Treatment | Bold | 4 | 13 | 5 | 18 |
| BT12 | Male | Treatment | Bold | 5 | 10 | 10 | 20 |
| BT12 | Male | Treatment | Bold | 6 | 9 | 12 | 21 |
| BT12 | Male | Treatment | Bold | 7 | 8 | 10 | 18 |
| BT12 | Male | Treatment | Bold | 8 | 9 | 9 | 18 |
| BT12 | Male | Treatment | Bold | 9 | 10 | 10 | 20 |
| BT12 | Male | Treatment | Bold | 10 | 11 | 9 | 20 |
| BC10 | Male | Control | Bold | 1 | 13 | 7 | 20 |
| BC10 | Male | Control | Bold | 2 | 12 | 7 | 19 |
| BC10 | Male | Control | Bold | 3 | 10 | 10 | 20 |
| BC10 | Male | Control | Bold | 4 | 10 | 10 | 20 |
| BC10 | Male | Control | Bold | 5 | 9 | 11 | 20 |
| BC10 | Male | Control | Bold | 6 | 11 | 8 | 19 |
| BC10 | Male | Control | Bold | 7 | 11 | 9 | 20 |
| BC10 | Male | Control | Bold | 8 | 8 | 11 | 19 |
| BC10 | Male | Control | Bold | 9 | 13 | 7 | 20 |
| BC10 | Male | Control | Bold | 10 | 10 | 8 | 18 |
| BC11 | Female | Control | Bold | 1 | 10 | 10 | 20 |
| BC11 | Female | Control | Bold | 2 | 11 | 9 | 20 |
| BC11 | Female | Control | Bold | 3 | 6 | 13 | 19 |
| BC11 | Female | Control | Bold | 4 | 10 | 9 | 19 |
| BC11 | Female | Control | Bold | 5 | 8 | 12 | 20 |
| BC11 | Female | Control | Bold | 6 | 10 | 10 | 20 |
| BC11 | Female | Control | Bold | 7 | 6 | 11 | 17 |
| BC11 | Female | Control | Bold | 8 | 9 | 11 | 20 |
| BC11 | Female | Control | Bold | 9 | 11 | 9 | 20 |
| BC11 | Female | Control | Bold | 10 | 12 | 8 | 20 |
| BC12 | Male | Control | Bold | 1 | 3 | 1 | 4 |
| BC12 | Male | Control | Bold | 2 | 14 | 2 | 16 |
| BC12 | Male | Control | Bold | 3 | 13 | 7 | 20 |
| BC12 | Male | Control | Bold | 4 | 3 | 3 | 6 |
| BC12 | Male | Control | Bold | 5 | 3 | 2 | 5 |
| BC12 | Male | Control | Bold | 6 | 6 | 5 | 11 |
| BC12 | Male | Control | Bold | 7 | 6 | 3 | 9 |
| BC12 | Male | Control | Bold | 8 | 6 | 4 | 10 |
| BC12 | Male | Control | Bold | 9 | 2 | 1 | 3 |
| BC12 | Male | Control | Shy | 10 | 3 | 0 | 3 |
| ST14 | Male | Treatment | Shy | 1 | 1 | 0 | 1 |
| ST14 | Male | Treatment | Shy | 2 | 1 | 0 | 1 |
| ST14 | Male | Treatment | Shy | 3 | 5 | 2 | 7 |
| ST14 | Male | Treatment | Shy | 4 | 6 | 3 | 9 |
| ST14 | Male | Treatment | Shy | 5 | 3 | 0 | 3 |
| ST14 | Male | Treatment | Shy | 6 | 15 | 3 | 18 |
| ST14 | Male | Treatment | Shy | 7 | 13 | 7 | 20 |
| ST14 | Male | Treatment | Shy | 8 | 19 | 1 | 20 |
| ST14 | Male | Treatment | Shy | 9 | 15 | 4 | 19 |
| ST14 | Male | Treatment | Shy | 10 | 15 | 5 | 20 |
| ST13 | Female | Treatment | Shy | 1 | 8 | 9 | 17 |
| ST13 | Female | Treatment | Shy | 2 | 7 | 1 | 8 |
| ST13 | Female | Treatment | Shy | 3 | 11 | 3 | 14 |
| ST13 | Female | Treatment | Shy | 4 | 13 | 2 | 15 |
| ST13 | Female | Treatment | Shy | 5 | 12 | 3 | 15 |
| ST13 | Female | Treatment | Shy | 6 | 10 | 5 | 15 |
| ST13 | Female | Treatment | Shy | 7 | 5 | 11 | 16 |
| ST13 | Female | Treatment | Shy | 8 | 12 | 5 | 17 |
| ST13 | Female | Treatment | Shy | 9 | 9 | 8 | 17 |
| ST13 | Female | Treatment | Shy | 10 | 8 | 8 | 16 |
| SC13 | Male | Control | Shy | 1 | 0 | 0 | 0 |
| SC13 | Male | Control | Shy | 2 | 0 | 0 | 0 |
| SC13 | Male | Control | Shy | 3 | 0 | 0 | 0 |
| SC13 | Male | Control | Shy | 4 | 0 | 0 | 0 |
| SC13 | Male | Control | Shy | 5 | 5 | 3 | 8 |
| SC13 | Male | Control | Shy | 6 | 0 | 0 | 0 |
| SC13 | Male | Control | Shy | 7 | 0 | 0 | 0 |
| SC13 | Male | Control | Shy | 8 | 1 | 0 | 1 |
| SC13 | Male | Control | Shy | 9 | 0 | 0 | 0 |
| SC13 | Male | Control | Shy | 10 | 0 | 0 | 0 |
| SC15 | Female | Control | Shy | 1 | 9 | 11 | 20 |
| SC15 | Female | Control | Shy | 2 | 5 | 12 | 17 |
| SC15 | Female | Control | Shy | 3 | 8 | 10 | 18 |
| SC15 | Female | Control | Shy | 4 | 6 | 10 | 16 |
| SC15 | Female | Control | Shy | 5 | 10 | 9 | 19 |
| SC15 | Female | Control | Shy | 6 | 4 | 9 | 13 |
| SC15 | Female | Control | Shy | 7 | 5 | 2 | 7 |
| SC15 | Female | Control | Shy | 8 | 7 | 5 | 12 |
| SC15 | Female | Control | Shy | 9 | 6 | 7 | 13 |
| SC15 | Female | Control | Bold | 10 | 2 | 7 | 9 |
| BT13 | Female | Treatment | Bold | 1 | 11 | 5 | 16 |
| BT13 | Female | Treatment | Bold | 2 | 9 | 7 | 16 |
| BT13 | Female | Treatment | Bold | 3 | 7 | 8 | 15 |
| BT13 | Female | Treatment | Bold | 4 | 13 | 7 | 20 |
| BT13 | Female | Treatment | Bold | 5 | 10 | 10 | 20 |
| BT13 | Female | Treatment | Bold | 6 | 7 | 10 | 17 |
| BT13 | Female | Treatment | Bold | 7 | 10 | 9 | 19 |
| BT13 | Female | Treatment | Bold | 8 | 12 | 6 | 18 |
| BT13 | Female | Treatment | Bold | 9 | 10 | 7 | 17 |
| BT13 | Female | Treatment | Bold | 10 | 8 | 8 | 16 |
| BT14 | Male | Treatment | Bold | 1 | 7 | 5 | 12 |
| BT14 | Male | Treatment | Bold | 2 | 6 | 6 | 12 |
| BT14 | Male | Treatment | Bold | 3 | 12 | 4 | 16 |
| BT14 | Male | Treatment | Bold | 4 | 6 | 7 | 13 |
| BT14 | Male | Treatment | Bold | 5 | 9 | 7 | 16 |
| BT14 | Male | Treatment | Bold | 6 | 14 | 5 | 19 |
| BT14 | Male | Treatment | Bold | 7 | 7 | 5 | 12 |
| BT14 | Male | Treatment | Bold | 8 | 8 | 9 | 17 |
| BT14 | Male | Treatment | Bold | 9 | 9 | 8 | 17 |
| BT14 | Male | Treatment | Bold | 10 | 6 | 6 | 12 |
| BT15 | Male | Treatment | Bold | 1 | 6 | 2 | 8 |
| BT15 | Male | Treatment | Bold | 2 | 2 | 10 | 12 |
| BT15 | Male | Treatment | Bold | 3 | 3 | 4 | 7 |
| BT15 | Male | Treatment | Bold | 4 | 5 | 2 | 7 |
| BT15 | Male | Treatment | Bold | 5 | 6 | 7 | 13 |
| BT15 | Male | Treatment | Bold | 6 | 6 | 3 | 9 |
| BT15 | Male | Treatment | Bold | 7 | 9 | 5 | 14 |
| BT15 | Male | Treatment | Bold | 8 | 12 | 6 | 18 |
| BT15 | Male | Treatment | Bold | 9 | 9 | 5 | 14 |
| BT15 | Male | Treatment | Bold | 10 | 8 | 7 | 15 |
| BC13 | Male | Control | Bold | 1 | 9 | 4 | 13 |
| BC13 | Male | Control | Bold | 2 | 9 | 6 | 15 |
| BC13 | Male | Control | Bold | 3 | 9 | 5 | 14 |
| BC13 | Male | Control | Bold | 4 | 7 | 8 | 15 |
| BC13 | Male | Control | Bold | 5 | 7 | 7 | 14 |
| BC13 | Male | Control | Bold | 6 | 8 | 5 | 13 |
| BC13 | Male | Control | Bold | 7 | 1 | 6 | 7 |
| BC13 | Male | Control | Bold | 8 | 8 | 12 | 20 |
| BC13 | Male | Control | Bold | 9 | 7 | 5 | 12 |
| BC13 | Male | Control | Bold | 10 | 11 | 8 | 19 |
| BC14 | Female | Control | Bold | 1 | 11 | 9 | 20 |
| BC14 | Female | Control | Bold | 2 | 13 | 7 | 20 |
| BC14 | Female | Control | Bold | 3 | 10 | 8 | 18 |
| BC14 | Female | Control | Bold | 4 | 8 | 8 | 16 |
| BC14 | Female | Control | Bold | 5 | 11 | 8 | 19 |
| BC14 | Female | Control | Bold | 6 | 11 | 7 | 18 |
| BC14 | Female | Control | Bold | 7 | 12 | 8 | 20 |
| BC14 | Female | Control | Bold | 8 | 10 | 9 | 19 |
| BC14 | Female | Control | Bold | 9 | 11 | 9 | 20 |
| BC14 | Female | Control | Shy | 10 | 10 | 10 | 20 |
| ST16 | Female | Treatment | Shy | 1 | 0 | 1 | 1 |
| ST16 | Female | Treatment | Shy | 2 | 6 | 14 | 20 |
| ST16 | Female | Treatment | Shy | 3 | 8 | 4 | 12 |
| ST16 | Female | Treatment | Shy | 4 | 4 | 6 | 10 |
| ST16 | Female | Treatment | Shy | 5 | 8 | 8 | 16 |
| ST16 | Female | Treatment | Shy | 6 | 2 | 9 | 11 |
| ST16 | Female | Treatment | Shy | 7 | 2 | 10 | 12 |
| ST16 | Female | Treatment | Shy | 8 | 8 | 9 | 17 |
| ST16 | Female | Treatment | Shy | 9 | 6 | 4 | 10 |
| ST16 | Female | Treatment | Shy | 10 | 9 | 7 | 16 |
| ST17 | Male | Treatment | Shy | 1 | 4 | 15 | 19 |
| ST17 | Male | Treatment | Shy | 2 | 1 | 15 | 16 |
| ST17 | Male | Treatment | Shy | 3 | 2 | 4 | 6 |
| ST17 | Male | Treatment | Shy | 4 | 4 | 2 | 6 |
| ST17 | Male | Treatment | Shy | 5 | 3 | 4 | 7 |
| ST17 | Male | Treatment | Shy | 6 | 3 | 2 | 5 |
| ST17 | Male | Treatment | Shy | 7 | 2 | 4 | 6 |
| ST17 | Male | Treatment | Shy | 8 | 1 | 4 | 5 |
| ST17 | Male | Treatment | Shy | 9 | 1 | 2 | 3 |
| ST17 | Male | Treatment | Shy | 10 | 5 | 2 | 7 |
| ST18 | Male | Treatment | Shy | 1 | 8 | 9 | 17 |
| ST18 | Male | Treatment | Shy | 2 | 7 | 8 | 15 |
| ST18 | Male | Treatment | Shy | 3 | 6 | 4 | 10 |
| ST18 | Male | Treatment | Shy | 4 | 9 | 3 | 12 |
| ST18 | Male | Treatment | Shy | 5 | 8 | 4 | 12 |
| ST18 | Male | Treatment | Shy | 6 | 5 | 9 | 14 |
| ST18 | Male | Treatment | Shy | 7 | 11 | 7 | 18 |
| ST18 | Male | Treatment | Shy | 8 | 5 | 7 | 12 |
| ST18 | Male | Treatment | Shy | 9 | 6 | 6 | 12 |
| ST18 | Male | Treatment | Shy | 10 | 9 | 10 | 19 |
| SC16 | Female | Control | Shy | 1 | 0 | 2 | 2 |
| SC16 | Female | Control | Shy | 2 | 3 | 5 | 8 |
| SC16 | Female | Control | Shy | 3 | 2 | 4 | 6 |
| SC16 | Female | Control | Shy | 4 | 4 | 11 | 15 |
| SC16 | Female | Control | Shy | 5 | 7 | 5 | 12 |
| SC16 | Female | Control | Shy | 6 | 9 | 9 | 18 |
| SC16 | Female | Control | Shy | 7 | 2 | 2 | 4 |
| SC16 | Female | Control | Shy | 8 | 6 | 8 | 14 |
| SC16 | Female | Control | Shy | 9 | 6 | 8 | 14 |
| SC16 | Female | Control | Shy | 10 | 5 | 6 | 11 |
| SC17 | Female | Control | Shy | 1 | 5 | 11 | 16 |
| SC17 | Female | Control | Shy | 2 | 7 | 8 | 15 |
| SC17 | Female | Control | Shy | 3 | 4 | 7 | 11 |
| SC17 | Female | Control | Shy | 4 | 10 | 9 | 19 |
| SC17 | Female | Control | Shy | 5 | 5 | 12 | 17 |
| SC17 | Female | Control | Shy | 6 | 10 | 6 | 16 |
| SC17 | Female | Control | Shy | 7 | 11 | 6 | 17 |
| SC17 | Female | Control | Shy | 8 | 7 | 11 | 18 |
| SC17 | Female | Control | Shy | 9 | 8 | 4 | 12 |
| SC17 | Female | Control | Bold | 10 | 6 | 11 | 17 |
| BT16 | Male | Treatment | Bold | 1 | 7 | 8 | 15 |
| BT16 | Male | Treatment | Bold | 2 | 9 | 8 | 17 |
| BT16 | Male | Treatment | Bold | 3 | 5 | 8 | 13 |
| BT16 | Male | Treatment | Bold | 4 | 4 | 11 | 15 |
| BT16 | Male | Treatment | Bold | 5 | 7 | 10 | 17 |
| BT16 | Male | Treatment | Bold | 6 | 9 | 5 | 14 |
| BT16 | Male | Treatment | Bold | 7 | 11 | 5 | 16 |
| BT16 | Male | Treatment | Bold | 8 | 8 | 4 | 12 |
| BT16 | Male | Treatment | Bold | 9 | 8 | 10 | 18 |
| BT16 | Male | Treatment | Bold | 10 | 9 | 6 | 15 |
| BT17 | Female | Treatment | Bold | 1 | 8 | 10 | 18 |
| BT17 | Female | Treatment | Bold | 2 | 4 | 8 | 12 |
| BT17 | Female | Treatment | Bold | 3 | 8 | 11 | 19 |
| BT17 | Female | Treatment | Bold | 4 | 4 | 10 | 14 |
| BT17 | Female | Treatment | Bold | 5 | 8 | 6 | 14 |
| BT17 | Female | Treatment | Bold | 6 | 10 | 5 | 15 |
| BT17 | Female | Treatment | Bold | 7 | 10 | 5 | 15 |
| BT17 | Female | Treatment | Bold | 8 | 8 | 6 | 14 |
| BT17 | Female | Treatment | Bold | 9 | 3 | 7 | 10 |
| BT17 | Female | Treatment | Bold | 10 | 8 | 5 | 13 |
| BT18 | Male | Treatment | Bold | 1 | 8 | 10 | 18 |
| BT18 | Male | Treatment | Bold | 2 | 9 | 11 | 20 |
| BT18 | Male | Treatment | Bold | 3 | 9 | 6 | 15 |
| BT18 | Male | Treatment | Bold | 4 | 7 | 11 | 18 |
| BT18 | Male | Treatment | Bold | 5 | 10 | 8 | 18 |
| BT18 | Male | Treatment | Bold | 6 | 8 | 6 | 14 |
| BT18 | Male | Treatment | Bold | 7 | 7 | 8 | 15 |
| BT18 | Male | Treatment | Bold | 8 | 6 | 10 | 16 |
| BT18 | Male | Treatment | Bold | 9 | 9 | 6 | 15 |
| BT18 | Male | Treatment | Bold | 10 | 10 | 10 | 20 |
| BC17 | Female | Control | Bold | 1 | 8 | 10 | 18 |
| BC17 | Female | Control | Bold | 2 | 3 | 13 | 16 |
| BC17 | Female | Control | Bold | 3 | 5 | 6 | 11 |
| BC17 | Female | Control | Bold | 4 | 6 | 11 | 17 |
| BC17 | Female | Control | Bold | 5 | 5 | 11 | 16 |
| BC17 | Female | Control | Bold | 6 | 7 | 8 | 15 |
| BC17 | Female | Control | Bold | 7 | 5 | 9 | 14 |
| BC17 | Female | Control | Bold | 8 | 8 | 10 | 18 |
| BC17 | Female | Control | Bold | 9 | 10 | 8 | 18 |
| BC17 | Female | Control | Bold | 10 | 9 | 11 | 20 |
| BC18 | Male | Control | Bold | 1 | 11 | 9 | 20 |
| BC18 | Male | Control | Bold | 2 | 8 | 9 | 17 |
| BC18 | Male | Control | Bold | 3 | 8 | 10 | 18 |
| BC18 | Male | Control | Bold | 4 | 12 | 7 | 19 |
| BC18 | Male | Control | Bold | 5 | 4 | 15 | 19 |
| BC18 | Male | Control | Bold | 6 | 8 | 11 | 19 |
| BC18 | Male | Control | Bold | 7 | 8 | 11 | 19 |
| BC18 | Male | Control | Bold | 8 | 11 | 9 | 20 |
| BC18 | Male | Control | Bold | 9 | 14 | 6 | 20 |
| BC18 | Male | Control | Shy | 10 | 5 | 11 | 16 |
| ST20 | Female | Treatment | Shy | 1 | 13 | 7 | 20 |
| ST20 | Female | Treatment | Shy | 2 | 11 | 9 | 20 |
| ST20 | Female | Treatment | Shy | 3 | 12 | 7 | 19 |
| ST20 | Female | Treatment | Shy | 4 | 12 | 8 | 20 |
| ST20 | Female | Treatment | Shy | 5 | 13 | 7 | 20 |
| ST20 | Female | Treatment | Shy | 6 | 12 | 8 | 20 |
| ST20 | Female | Treatment | Shy | 7 | 7 | 13 | 20 |
| ST20 | Female | Treatment | Shy | 8 | 12 | 7 | 19 |
| ST20 | Female | Treatment | Shy | 9 | 10 | 10 | 20 |
| ST20 | Female | Treatment | Shy | 10 | 8 | 12 | 20 |
| SC20 | Male | Control | Shy | 1 | 5 | 13 | 18 |
| SC20 | Male | Control | Shy | 2 | 3 | 9 | 12 |
| SC20 | Male | Control | Shy | 3 | 6 | 11 | 17 |
| SC20 | Male | Control | Shy | 4 | 6 | 13 | 19 |
| SC20 | Male | Control | Shy | 5 | 9 | 11 | 20 |
| SC20 | Male | Control | Shy | 6 | 8 | 10 | 18 |
| SC20 | Male | Control | Shy | 7 | 5 | 14 | 19 |
| SC20 | Male | Control | Shy | 8 | 11 | 9 | 20 |
| SC20 | Male | Control | Shy | 9 | 14 | 6 | 20 |
| SC20 | Male | Control | Shy | 10 | 8 | 11 | 19 |
| SC21 | Male | Control | Shy | 1 | 11 | 3 | 14 |
| SC21 | Male | Control | Shy | 2 | 6 | 12 | 18 |
| SC21 | Male | Control | Shy | 3 | 5 | 15 | 20 |
| SC21 | Male | Control | Shy | 4 | 8 | 12 | 20 |
| SC21 | Male | Control | Shy | 5 | 14 | 6 | 20 |
| SC21 | Male | Control | Shy | 6 | 11 | 9 | 20 |
| SC21 | Male | Control | Shy | 7 | 12 | 7 | 19 |
| SC21 | Male | Control | Shy | 8 | 11 | 8 | 19 |
| SC21 | Male | Control | Shy | 9 | 10 | 8 | 18 |
| SC21 | Male | Control | Shy | 10 | 14 | 7 | 21 |
| SC22 | Female | Control | Shy | 1 | 6 | 10 | 16 |
| SC22 | Female | Control | Shy | 2 | 8 | 11 | 19 |
| SC22 | Female | Control | Shy | 3 | 5 | 13 | 18 |
| SC22 | Female | Control | Shy | 4 | 10 | 10 | 20 |
| SC22 | Female | Control | Shy | 5 | 7 | 6 | 13 |
| SC22 | Female | Control | Shy | 6 | 7 | 9 | 16 |
| SC22 | Female | Control | Shy | 7 | 12 | 8 | 20 |
| SC22 | Female | Control | Shy | 8 | 5 | 14 | 19 |
| SC22 | Female | Control | Shy | 9 | 5 | 11 | 16 |
| SC22 | Female | Control | Bold | 10 | 11 | 8 | 19 |
| BC23 | Female | Control | Bold | 1 | 9 | 4 | 13 |
| BC23 | Female | Control | Bold | 2 | 11 | 7 | 18 |
| BC23 | Female | Control | Bold | 3 | 13 | 7 | 20 |
| BC23 | Female | Control | Bold | 4 | 11 | 6 | 17 |
| BC23 | Female | Control | Bold | 5 | 9 | 6 | 15 |
| BC23 | Female | Control | Bold | 6 | 10 | 6 | 16 |
| BC23 | Female | Control | Bold | 7 | 14 | 4 | 18 |
| BC23 | Female | Control | Bold | 8 | 13 | 7 | 20 |
| BC23 | Female | Control | Bold | 9 | 11 | 2 | 13 |
| BC23 | Female | Control | Bold | 10 | 9 | 6 | 15 |
| BC24 | Male | Control | Bold | 1 | 2 | 9 | 11 |
| BC24 | Male | Control | Bold | 2 | 12 | 6 | 18 |
| BC24 | Male | Control | Bold | 3 | 12 | 6 | 18 |
| BC24 | Male | Control | Bold | 4 | 6 | 9 | 15 |
| BC24 | Male | Control | Bold | 5 | 11 | 11 | 22 |
| BC24 | Male | Control | Bold | 6 | 12 | 10 | 22 |
| BC24 | Male | Control | Bold | 7 | 11 | 6 | 17 |
| BC24 | Male | Control | Bold | 8 | 14 | 7 | 21 |
| BC24 | Male | Control | Bold | 9 | 9 | 8 | 17 |
| BC24 | Male | Control | Bold | 10 | 12 | 10 | 22 |
| BC19 | Female | Control | Bold | 1 | 11 | 3 | 14 |
| BC19 | Female | Control | Bold | 2 | 8 | 8 | 16 |
| BC19 | Female | Control | Bold | 3 | 14 | 7 | 21 |
| BC19 | Female | Control | Bold | 4 | 6 | 10 | 16 |
| BC19 | Female | Control | Bold | 5 | 8 | 9 | 17 |
| BC19 | Female | Control | Bold | 6 | 12 | 8 | 20 |
| BC19 | Female | Control | Bold | 7 | 11 | 9 | 20 |
| BC19 | Female | Control | Bold | 8 | 7 | 6 | 13 |
| BC19 | Female | Control | Bold | 9 | 13 | 11 | 24 |
| BC19 | Female | Control | Bold | 10 | 12 | 7 | 19 |
| BC20 | Female | Control | Bold | 1 | 11 | 8 | 19 |
| BC20 | Female | Control | Bold | 2 | 9 | 10 | 19 |
| BC20 | Female | Control | Bold | 3 | 12 | 7 | 19 |
| BC20 | Female | Control | Bold | 4 | 9 | 11 | 20 |
| BC20 | Female | Control | Bold | 5 | 16 | 2 | 18 |
| BC20 | Female | Control | Bold | 6 | 10 | 5 | 15 |
| BC20 | Female | Control | Bold | 7 | 13 | 5 | 18 |
| BC20 | Female | Control | Bold | 8 | 5 | 12 | 17 |
| BC20 | Female | Control | Bold | 9 | 11 | 6 | 17 |
| BC20 | Female | Control | Bold | 10 | 12 | 8 | 20 |
| BT20 | Female | Treatment | Bold | 1 | 14 | 6 | 20 |
| BT20 | Female | Treatment | Bold | 2 | 14 | 5 | 19 |
| BT20 | Female | Treatment | Bold | 3 | 6 | 10 | 16 |
| BT20 | Female | Treatment | Bold | 4 | 9 | 9 | 18 |
| BT20 | Female | Treatment | Bold | 5 | 10 | 8 | 18 |
| BT20 | Female | Treatment | Bold | 6 | 8 | 11 | 19 |
| BT20 | Female | Treatment | Bold | 7 | 16 | 4 | 20 |
| BT20 | Female | Treatment | Bold | 8 | 7 | 13 | 20 |
| BT20 | Female | Treatment | Bold | 9 | 10 | 9 | 19 |
| BT20 | Female | Treatment | Bold | 10 | 12 | 7 | 19 |
| BC22 | Male | Control | Bold | 1 | 3 | 7 | 10 |
| BC22 | Male | Control | Bold | 2 | 7 | 9 | 16 |
| BC22 | Male | Control | Bold | 3 | 4 | 6 | 10 |
| BC22 | Male | Control | Bold | 4 | 7 | 13 | 20 |
| BC22 | Male | Control | Bold | 5 | 9 | 11 | 20 |
| BC22 | Male | Control | Bold | 6 | 8 | 8 | 16 |
| BC22 | Male | Control | Bold | 7 | 4 | 6 | 10 |
| BC22 | Male | Control | Bold | 8 | 6 | 13 | 19 |
| BC22 | Male | Control | Bold | 9 | 2 | 7 | 9 |
| BC22 | Male | Control | Shy | 10 | 7 | 7 | 14 |
| SC23 | Female | Control | Shy | 1 | 1 | 4 | 5 |
| SC23 | Female | Control | Shy | 2 | 11 | 9 | 20 |
| SC23 | Female | Control | Shy | 3 | 8 | 12 | 20 |
| SC23 | Female | Control | Shy | 4 | 13 | 7 | 20 |
| SC23 | Female | Control | Shy | 5 | 15 | 5 | 20 |
| SC23 | Female | Control | Shy | 6 | 12 | 8 | 20 |
| SC23 | Female | Control | Shy | 7 | 14 | 6 | 20 |
| SC23 | Female | Control | Shy | 8 | 11 | 8 | 19 |
| SC23 | Female | Control | Shy | 9 | 12 | 6 | 18 |
| SC23 | Female | Control | Shy | 10 | 12 | 7 | 19 |
| SC25 | Male | Control | Shy | 1 | 5 | 6 | 11 |
| SC25 | Male | Control | Shy | 2 | 6 | 7 | 13 |
| SC25 | Male | Control | Shy | 3 | 5 | 10 | 15 |
| SC25 | Male | Control | Shy | 4 | 4 | 8 | 12 |
| SC25 | Male | Control | Shy | 5 | 4 | 5 | 9 |
| SC25 | Male | Control | Shy | 6 | 6 | 5 | 11 |
| SC25 | Male | Control | Shy | 7 | 10 | 9 | 19 |
| SC25 | Male | Control | Shy | 8 | 6 | 7 | 13 |
| SC25 | Male | Control | Shy | 9 | 7 | 6 | 13 |
| SC25 | Male | Control | Shy | 10 | 4 | 6 | 10 |
| SC26 | Female | Control | Shy | 1 | 1 | 0 | 1 |
| SC26 | Female | Control | Shy | 2 | 0 | 0 | 0 |
| SC26 | Female | Control | Shy | 3 | 1 | 0 | 1 |
| SC26 | Female | Control | Shy | 4 | 0 | 1 | 1 |
| SC26 | Female | Control | Shy | 5 | 1 | 2 | 3 |
| SC26 | Female | Control | Shy | 6 | 1 | 0 | 1 |
| SC26 | Female | Control | Shy | 7 | 1 | 0 | 1 |
| SC26 | Female | Control | Shy | 8 | 0 | 0 | 0 |
| SC26 | Female | Control | Shy | 9 | 1 | 1 | 2 |
| SC26 | Female | Control | Shy | 10 | 1 | 1 | 2 |
| SC27 | Male | Control | Shy | 1 | 4 | 4 | 8 |
| SC27 | Male | Control | Shy | 2 | 7 | 11 | 18 |
| SC27 | Male | Control | Shy | 3 | 11 | 9 | 20 |
| SC27 | Male | Control | Shy | 4 | 10 | 9 | 19 |
| SC27 | Male | Control | Shy | 5 | 7 | 10 | 17 |
| SC27 | Male | Control | Shy | 6 | 4 | 10 | 14 |
| SC27 | Male | Control | Shy | 7 | 5 | 9 | 14 |
| SC27 | Male | Control | Shy | 8 | 10 | 4 | 14 |
| SC27 | Male | Control | Shy | 9 | 8 | 10 | 18 |
| SC27 | Male | Control | Shy | 10 | 9 | 4 | 13 |
| SC28 | Female | Control | Shy | 1 | 11 | 9 | 20 |
| SC28 | Female | Control | Shy | 2 | 12 | 8 | 20 |
| SC28 | Female | Control | Shy | 3 | 10 | 10 | 20 |
| SC28 | Female | Control | Shy | 4 | 10 | 9 | 19 |
| SC28 | Female | Control | Shy | 5 | 6 | 12 | 18 |
| SC28 | Female | Control | Shy | 6 | 12 | 8 | 20 |
| SC28 | Female | Control | Shy | 7 | 7 | 12 | 19 |
| SC28 | Female | Control | Shy | 8 | 10 | 10 | 20 |
| SC28 | Female | Control | Shy | 9 | 14 | 3 | 17 |
| SC28 | Female | Control | Shy | 10 | 6 | 13 | 19 |
| ST22 | Female | Treatment | Shy | 1 | 0 | 5 | 5 |
| ST22 | Female | Treatment | Shy | 2 | 0 | 10 | 10 |
| ST22 | Female | Treatment | Shy | 3 | 0 | 6 | 6 |
| ST22 | Female | Treatment | Shy | 4 | 1 | 3 | 4 |
| ST22 | Female | Treatment | Shy | 5 | 1 | 3 | 4 |
| ST22 | Female | Treatment | Shy | 6 | 1 | 2 | 3 |
| ST22 | Female | Treatment | Shy | 7 | 2 | 3 | 5 |
| ST22 | Female | Treatment | Shy | 8 | 2 | 3 | 5 |
| ST22 | Female | Treatment | Shy | 9 | 3 | 12 | 15 |
| ST22 | Female | Treatment | Shy | 10 | 4 | 7 | 11 |
| ST23 | Female | Treatment | Shy | 1 | 3 | 2 | 5 |
| ST23 | Female | Treatment | Shy | 2 | 3 | 0 | 3 |
| ST23 | Female | Treatment | Shy | 3 | 4 | 4 | 8 |
| ST23 | Female | Treatment | Shy | 4 | 8 | 3 | 11 |
| ST23 | Female | Treatment | Shy | 5 | 5 | 9 | 14 |
| ST23 | Female | Treatment | Shy | 6 | 5 | 7 | 12 |
| ST23 | Female | Treatment | Shy | 7 | 11 | 9 | 20 |
| ST23 | Female | Treatment | Shy | 8 | 7 | 4 | 11 |
| ST23 | Female | Treatment | Shy | 9 | 5 | 7 | 12 |
| ST23 | Female | Treatment | Shy | 10 | 8 | 6 | 14 |
| ST24 | Male | Treatment | Shy | 1 | 6 | 6 | 12 |
| ST24 | Male | Treatment | Shy | 2 | 4 | 2 | 6 |
| ST24 | Male | Treatment | Shy | 3 | 2 | 5 | 7 |
| ST24 | Male | Treatment | Shy | 4 | 3 | 1 | 4 |
| ST24 | Male | Treatment | Shy | 5 | 6 | 2 | 8 |
| ST24 | Male | Treatment | Shy | 6 | 6 | 7 | 13 |
| ST24 | Male | Treatment | Shy | 7 | 6 | 7 | 13 |
| ST24 | Male | Treatment | Shy | 8 | 9 | 6 | 15 |
| ST24 | Male | Treatment | Shy | 9 | 12 | 5 | 17 |
| ST24 | Male | Treatment | Shy | 10 | 12 | 7 | 19 |
| *N* = 76 |  |  |  |  |  |  |  |
